# Low temporal dynamics of mycosporine‐like amino acids in benthic cyanobacteria from an alpine lake

**DOI:** 10.1111/fwb.13627

**Published:** 2020-10-15

**Authors:** Nadine Werner, Maria Orfanoudaki, Anja Hartmann, Markus Ganzera, Ruben Sommaruga

**Affiliations:** ^1^ Department of Ecology University of Innsbruck Innsbruck Austria; ^2^ Institute of Pharmacy Pharmacognosy University of Innsbruck Innsbruck Austria

**Keywords:** aplysiapalythine, Benthos, biofilm, epilithon, mountain lakes, UV radiation

## Abstract

1. Cyanobacteria are one of the oldest organisms on Earth and they originated at a time when damaging ultraviolet (UV) C radiation still reached the surface. Their long evolution led to several adaptations to avoid deleterious effects caused by exposure to solar UV radiation. Synthesis of sunscreen substances, such as mycosporine‐like amino acids (MAAs), allows them to photosynthesise with reduced risk of cell damage. The interplay of solar UV radiation and MAAs is well documented for cyanobacteria in the plankton realm, but little is known for those in the benthic realm, particularly of clear alpine lakes.
2. Here, we assessed the temporal dynamics of MAAs in the benthic algal community of one clear alpine lake dominated by cyanobacteria during the ice‐free season and along a depth gradient using state‐of‐the‐art analytical methods (high‐performance liquid chromatography, nuclear magnetic resonance, liquid chromatography–mass spectrometry). We differentiated between the epilithic cyanobacterial community and the overlying loosely attached filamentous cyanobacteria, as we expected they will have an important shielding/shading effect on the former. We hypothesised that in contrast to the case of phytoplankton, benthic cyanobacteria will show less pronounced temporal changes in MAAs concentration in response to changes in solar UV exposure.
3. Three UV‐absorbing substances were present in both types of communities, whereby all were unknown. The chemical structure of the dominant unknown substance (maximum absorption at 334 nm) resulted in the identification of a novel MAA that we named aplysiapalythine‐D for its similarity to the previously described aplysiapalythine‐C.
4. Chlorophyll‐*a*‐specific MAA concentrations for epilithic and filamentous cyanobacteria showed a significant decrease with depth, although only traces were found in the former community. The temporal dynamics in MAA concentrations of filamentous cyanobacteria showed no significant variations during the ice‐free season.
5. Our result on the low temporal MAA dynamics agrees with the reduced growth rates of benthic cyanobacteria reported for cold ecosystems. The permanent presence of this community, which is adapted to the high UV levels characteristic of clear alpine lakes, probably represents the most important primary producers of these ecosystems.

## 1 INTRODUCTION

Cyanobacteria were the first oxygenic photosynthetic microorganisms on Earth, and they contributed significantly to the build‐up of oxygen in the atmosphere (Fischer, 2008). Due to their existence before the development of the ozone layer (Sinha et al., 1996), cyanobacteria have adapted to damaging levels of incident solar ultraviolet radiation (UVR: 280–400 nm) and are able to inhabit extreme environments (Mushir & Fatma, 2012). They have a cosmopolitan distribution and consequently they are found under a wide range of environmental conditions (Gao & Garcia‐Pichel, 2011; Sinha et al., 1996).

Alpine lakes are harsh environments where UVR usually constitutes a stress factor (Sommaruga, 2001). This is because incident UVR levels increase with elevation and, for example, in the Alps, this factor is on average 9% within the UV‐A (315–400 nm) and 18% within the UV‐B (280–315 nm) radiation range per 1,000 m and under clear sky conditions (Blumthaler, Ambach, & Rehwald, 1992). Furthermore, owing to the location of these lakes above treeline and thus, with little or no vegetation soil cover, they contain low concentrations of dissolved organic matter. Because dissolved organic matter, particularly its coloured or chromophoric fraction, has a high absorbing capacity in the UV range, it functions as a shield against those wavelengths. In clear alpine lakes, however, it offers only little protection against UVR (Sommaruga, 2001; Vinebrooke & Leavitt, 1996) and so these wavelengths can penetrate very deep with still significant levels (Laurion et al., 2000). Thus, the two major photosynthetic communities (in terms of biomass) in clear alpine lakes, namely phytoplankton and benthic algae/cyanobacteria (Lambert & Cattaneo, 2008), need to be adapted to this stress factor to be able to colonise them.

The damaging effects of UVR on living organisms in alpine lakes are relatively well studied (Doyle et al., 2005; Sommaruga, 2001; Tartarotti et al., 2014; Vinebrooke & Leavitt, 1996). Photosynthetically active communities need to balance potential negative effects caused by UVR with the required exposure to wavelengths needed for photosynthesis (i.e. photosynthetically active radiation or PAR: 400–700 nm). For this reason, they have adapted by evolving different strategies to counteract the adverse effects of solar UVR (Mushir & Fatma, 2012; Rastogi et al., 2014; Vincent & Roy, 1993). Vertical migration of organisms to low UV refuges in deeper layers of the lake is one of them (Tartarotti et al., 2001; Vinebrooke & Leavitt, 1996). However, as most benthic primary producers are non‐motile (Vinebrooke & Leavitt, 1996), the synthesis of sunscreen compounds such as mycosporine‐like amino acids (MAAs) and scytonemin provide their first line of defence against UVR (Cockell & Knowland, 1999; Gao & Garcia‐Pichel, 2011).

Mycosporine‐like amino acids are colourless, water soluble substances, characterised by a 5‐hydroxy‐5‐hydroxymethyl‐cyclohex‐1,2‐ene ring with a methoxy‐substituent (Gao & Garcia‐Pichel, 2011), with narrow absorption bands (Garcia‐Pichel & Castenholz, 1993) and absorbance maxima ranging from 309–360 nm (Cockell & Knowland, 1999; Gao & Garcia‐Pichel, 2011; Pathak et al., 2019). The C3 position of the ring structure of MAAs is commonly substituted with an amino compound, while the C1 position is substituted with either an oxo or an imino moiety (Gao & Garcia‐Pichel, 2011).

In planktonic communities, both large temporal and spatial variations of MAA concentrations have been reported. These studies showed that, for example, MAA concentrations decrease with depth during the ice‐free period (Sommaruga & Garcia‐Pichel, 1999) and that the highest mean MAA concentration is observed in the summer months, suggesting an adaptation to this stress factor (Tartarotti & Sommaruga, 2006). Similar knowledge for benthic primary producers of alpine lakes is not available in contrast to few other studies for the high Arctic (Bonilla et al., 2005; Quesada et al., 1999). In fact, lake phytoplankton studies largely exceed those on lake periphyton (Cantonati & Lowe, 2014), leading to a gap of knowledge on their ecology (Vadeboncoeur et al., 2002). This gap is even more evident for alpine lakes. One of the few studies on benthic cyanobacteria in alpine lakes (Rott & Pernegger, 1994) identified four depth zones with different cyanobacterial taxonomic composition. These authors also highlighted the occurrence of a high biomass of cyanobacteria, but with low temporal turnover. In the same lake, MAAs have been primarily analysed in the planktonic realm (Sommaruga & Garcia‐Pichel, 1999; Tartarotti & Sommaruga, 2006), even though in the former study the presence of UV‐absorbing compounds in littoral benthic cyanobacteria was reported.

In the present study, the temporal variation in the concentration and composition of MAAs in benthic cyanobacteria of an alpine lake was studied. We expected to find low temporal variations in MAA concentration during the ice‐free period. However, a decrease of MAA concentration with depth, similar to what is observed for planktonic communities (Sommaruga, 2001; Sommaruga & Garcia‐Pichel, 1999), is anticipated due to the typical attenuation of UV radiation with depth (Laurion et al., 2000). Additionally, considering that filamentous cyanobacteria will cause a shading effect on the underlying epilithic biofilm, we expected to find higher concentrations of MAAs in the former community.

## 2 METHODS

### 2.1 Study site and sampling

The oligotrophic alpine lake Gossenköllesee (2,417 m above sea level, Central Alps, Tyrol, Austria; 47.229667°N, 11.013938°E) was sampled at *c*. 2‐week intervals during the ice‐free period (July–October) in 2018. The lake has a maximum depth of 9.9 m and is mainly fed by surface runoff and groundwater sources (i.e. seepage lake). The lake has a high UV transparency with 10% of surface UV‐B and 33% of surface UV‐A radiation reaching the bottom at 9.9 m depth (Laurion et al., 2000; Sommaruga & Garcia‐Pichel, 1999).

Sampling was conducted by two SCUBA divers and consisted of three stones per depth collected at 0.4, 2.0, 4.0, and 7.7 m. These depths correspond with the cyanobacterial taxonomic zonation described for Gossenköllesee by Rott and Pernegger (1994). The epilithic biofilm was brushed from the stones with a toothbrush and screened through a 100‐µm net. The screening led to a partitioning of the samples into a loosely filamentous cyanobacteria (>100 µm) and an underlying epilithic fraction (<100 µm).

The filamentous cyanobacteria were placed in separate Eppendorf safe lock tubes and stored at −80°C until further analysis. The underlying epilithic community was filtered through Whatman GF/F filters (Maidstone, UK) separately for MAA and chlorophyll‐*a* (Chl‐*a*) before storage at −80°C. Samples for MAA and Chl‐*a* analysis in the filamentous cyanobacteria were lyophilised (LMC Gamma 1–20, Christ, Osterode am Harz, Germany) before extraction. For each analysis, the three stones were considered as replicates.

### 2.2 Photosynthetically active radiation and temperature measurements

Photosynthetically active radiation (400–700 nm) and water temperature were monitored at three depths (0.5, 2, and 8 m) from 22 June 2018 (day of year or doy 173) to 21 October 2018 (doy 294) at 15 min intervals using submersible loggers (miniPAR, Precision Measurement Engineering, Vista CA, USA and TidBit v2, Onset, Bourne, MD, USA, respectively). The loggers were placed on a moored line at the centre of the lake.

### 2.3 Chlorophyll‐*a* analysis

Samples were extracted in 90% alkaline acetone (addition of NH_4_OH) and ultra‐sonicated (Sonopuls UW 60, Bandelin, Berlin, Germany) for 2 min at 50% power. Afterwards, the samples were left at 4°C overnight before the spectrophotometric measurements took place (U‐2000, Hitachi, Tokyo, Japan). To calculate the Chl‐*a* concentration, the equation of Lorenzen (1967) was used and expressed as µg/mg dry weight for filaments and as µg/cm^2^ for epilithon samples.

### 2.4 Mycosporine‐like amino acid analysis

Mycosporine‐like amino acids were extracted in 25% aqueous methanol in a water bath for 2 hr at 45°C after ultra‐sonification (Sonopuls HD 2070, Bandelin, Berlin, Germany) for 2 min at 50% power and then stored at −80°C overnight (Tartarotti, & Sommaruga, 2002). After the samples were completely thawed and centrifugated for 20 min at 16,000 g at 10°C (Centrifuge 5,415 R, Eppendorf, Hamburg, Germany), 100 µl were transferred into chromatography‐vials with inserts and analysed by high‐performance liquid chromatography (HPLC; Dionex, Vienna, Austria). The mobile phase during isocratic analysis was 25% aqueous methanol plus 0.1% acetic acid (Tartarotti & Sommaruga, 2002). The MAAs were compared with secondary standards of the marine red alga *Porphyra* sp. through co‐chromatography. Mycosporine‐like amino acid concentrations were normalised to the content of Chl‐*a*, which was analysed in parallel samples. As the chemical structure and absorption maximum of the dominant and later‐identified new MAA, aplysiapalythine (AP)‐D, were similar to that of asterina‐330, and the available amount of the former was insufficient for establishing a calibration curve, the molar extinction coefficient of asterina‐330 (43,800 M^−1^ cm^−1^; Kamio et al., 2011) was used for its quantification. The concentrations of two other unknown MAAs (λ_max_ = 320 nm and 346 nm) were calculated using the mean molar extinction coefficient of 40,000 M^−1^ cm^−1^ and a mass of 300 Da (Gacesa et al., 2018; Wada et al., 2015).

### 2.5 Chemical characterisation of unknown MAAs

Nuclear magnetic resonance (NMR) measurements were conducted on a Bruker Avance II 600 spectrometer (Karlsruhe, Germany) operating at 600.19 (^1^H) and 150.91 MHz (^13^C). The isolated compound was dissolved in deuterated water from Eurisotop (Saint Aubin, France) containing tetramethylsilane as internal standard. High‐resolution mass spectra were measured with a micrOTOF‐Q II mass spectrometer (Bruker‐Daltonics, Bremen, Germany), whereas low‐resolution mass spectra were recorded on an Agilent InfinityLab LC/MSD System, comprising an Agilent 1260 HPLC, equipped with binary pump, autosampler, column oven and photodiode array detector (Santa Clara, CA, USA). For the purification of compounds, a semi‐preparative HPLC from Dionex (UltiMate 3000; Thermo, Waltham, MA, USA) was used comprising a P580 pump, an ASI 100 automated sample injector, an UVD 170 U detector and a fraction collector.

Solvents for analytical measurements were of pro analysis quality (Merck, Germany). The samples were extracted thrice in an ultrasonic bath (Bandelin Sonorex 35 KHz, Berlin, Germany) for 15 min using pure methanol, followed by three extractions with water (ultrapure water Sartorius arium^®^ 611 UV, Göttingen, Germany). High‐performance liquid chromatography analyses indicated that both extracts contained MAAs; therefore, they were combined for further fractionation. They were evaporated under reduced pressure, suspended in distilled water, and then partitioned with ethyl acetate, which was distilled before use. The ethyl acetate fraction was disposed and the water phase (72 mg dry weight) was subjected to semi‐preparative HPLC using a Synergi Polar‐RP column (250 × 10 mm, 4 µm; Phenomenex, Torrance, CA, USA). The mobile phase comprised of 0.25% (v/v) formic acid in water (A) and methanol (B), and the following gradient was used: 0 min: 2% B, 5 min: 5% B, 25 min: 10% B, 30 min: 98% B, 30.1–40 min: 2% B. After a final purification step (water/methanol, 3/1, v/v) on Sephadex LH‐20 (Sigma, USA) material, the new MAA (1.5 mg) was obtained.

### 2.6 Data analyses

All data analyses were run in R (Version 3.5.1) using the following packages: graphical illustrations, as well as integrated PAR levels, were conducted using tidyverse (Wickham et al., 2019), ggpubr (v0.2; Kassambara, 2020 https://cran.r‐project.org/web/packages/ggpubr/index.html), as well as Hmisc (v4.4‐0; Harrell, 2020 https://cran.r‐project.org/web/packages/Hmisc/Hmisc.pdf). For correlation analyses, the packages corrplot (v0.84; Wei et al., 2017, https://cran.r‐project.org/web/packages/corrplot/corrplot.pdf) and corrr (v0.3.1; Ruiz et al., 2019; https://www.rdocumentation.org/packages/corrr/versions/0.3.1) were used. Data are reported as mean ± 1 standard deviation and with a level of significance of *p* < 0.05.

## 3 RESULTS

### 3.1 Photosynthetically active radiation and water temperature

Water temperature ranged from 7.5 to 16.1°C at 0.5 m depth, from 7.5 to 14.5°C at 4.0 m, and from 4.9 to 8.8°C at 8.0 m (Figure [Link], [Link]). Mixing of the water column began on 3 October corresponding to doy 276, when the same temperature was measured at all three depths. Daily integrated PAR values for the three depths (Figure S2) ranged from 4.34 to 47.51 mol m^−2^ d^−1^ at 0.5 m, from 2.37 to 23.49 mol m^−2^ d^−1^ at 4 m, and from 1.11 to 13.12 mol m^−2^ d^−1^ at 8 m (Figure S1). The highest integrated PAR value was observed on 9 July 2018 (doy 190; 0.5 m: 39.86, 4 m: 18.86, 8 m: 9.16 mol m^−2^ d^−1^) and the lowest on doy 222 (0.5 m: 8.58, 4 m: 4.13, and 8 m: 2.15 mol m^−2^ d^−1^).

### 3.2 Structure elucidation of a novel MAA

**FIGURE 1:**
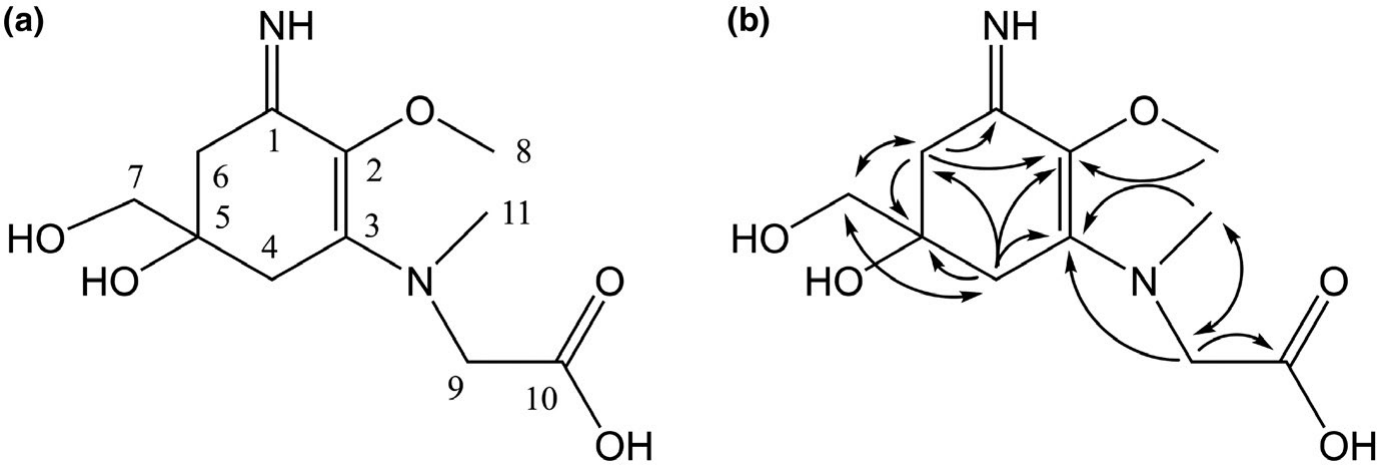
Chemical structure of the novel mycosporine‐like amino acid as elucidated by nuclear magnetic resonance and high‐resolution mass spectrometry (a) and key heteronuclear multiple bond coherence (^1^H → ^13^C) correlations of the novel mycosporine‐like amino acid (b)

A comparison of the possibly new MAAs with reference standards by HPLC showed no agreement. The dominant molecule in all samples with a maximum of absorption at 334 nm (Figure 1a) was therefore analysed by NMR after its isolation. It showed characteristic NMR shifts (Table 1, Figures [Link], [Link], [Link], [Link], [Link]) indicating the presence of a cyclohexenimine‐type MAA scaffold (C‐1 at *δ*_C_ 166.2 and C‐3 at *δ*_C_ 163.1). The side chain was identified as glycine based on a heteronuclear multiple bond coherence (HMBC) correlation (Figure 1b) of the methylene at position 9 (*δ*_H_ 4.46) and the carbonyl group at *δ* **_C_** 177.9 (C‐10), and by comparison with literature values (Tu et al., 2006). Its position was confirmed by a long‐range correlation visible in the HMBC spectrum (H‐9 at *δ*_H_ 4.46 to C‐3 at *δ*_C_ 163.1). Furthermore, protons of the methylene at position 9 (*δ*_H_ 4.46) showed a correlation in the HMBC spectrum with the N‐methyl group at *δ*_C_ 45.3 (Figure 1b), indicating that, compared to palythine, the isolated MAA possessed an extra methyl group at the N atom attached to C‐3. The isolated compound was finally identified as a novel MAA, N‐(5‐hydroxy‐5‐(hydroxymethyl)‐3‐imino‐2‐methoxycyclohex‐1‐en‐1‐yl)‐N‐methylglycine, with the molecular formula C_11_H_18_N_2_O_5_ (high‐resolution mass spectrometry data: [M + H]^+^ = 259.1244 (Calculated mass or Calcd for C_11_H_19_N_2_O_5:_ 259.1224), [M + Na]^+^ = 281.1068 (Calcd for C_11_H_18_N_2_O_5_: 281.1113), [2M + Na]^+^ = 539.2278 (Calcd for C_11_H_18_N_2_O_5_: 539.2329, Figure S8), and was given the name AP‐D.

**TABLE 1:** Nuclear magnetic resonance (NMR) data of the novel mycosporine‐like amino acid

Position	^1^H‐NMR ^a^	^13^C‐NMR ^a^
	*δ*_H_ (*J* in Hz)	*δ*_C_, type
1		166.2, C
2		129.1, C
3		163.1, C
4	2.92, d (17.2)	40.6, CH_2_
5		73.5, C
6	2.69, d (17.2) 2.94, d (17.2)	38.8, CH_2_
7	3.59, s	70.3, CH_2_
8	3.53, s	63.3, CH_3_
9	4.43, d (17.4) 4.49, d (17.4)	61.8, CH_2_
10		177.9, C
11	3.26, s	45.3, CH_3_

^a^ Recorded in D_2_O (600 MHz).

Apart from this new MAA, presumably two additional MAAs were also present in the combined methanolic and aqueous extract. They were tentatively assigned as such because of their typical UV spectra (Figures S9 and S10); however, due to low concentrations in the extract (Figure S11) their isolation was not possible. The absorption maxima of the unidentified MAA at 10.6 min was 325 nm (Figure S9) and its dominant m/z values were 438 ([M + H]^+^) and 436 ([M‐H]^‐^; Figures S12 and S13), whereas absorption maxima and molecular mass of the second compound at 14.9 min were 320 nm and 421, respectively (Figures S10, S14 and S15). The mass spectrometry of both unidentified MAAs revealed a prominent fragment ion corresponding to the loss of a hexose.

### 3.3 Mycosporine‐like amino acid concentration along the depth gradient

**FIGURE 2:**
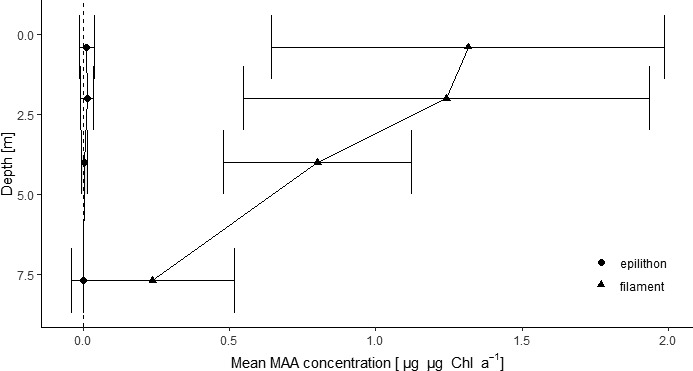
Chlorophyll‐*a*‐specific mycosporine‐like amino acid (MAA) concentration for the study period between 7 July and 18 October 2018 in cyanobacterial filaments (filament) and in the epilithic community (epilithon) at 0.4, 2, 4, and 8 m depth

Mean Chl‐*a*‐specific MAA concentration of the novel MAA ranged from 0.0005 to 1.32 µg/µg Chl‐*a* in the depth gradient (Figure 2). The MAA concentration in both filamentous and the underlying epilithic cyanobacteria decreased with depth. The filamentous cyanobacteria showed on average 100‐, 93‐, 142‐, and 485‐fold higher values than in the underlying epilithic community at 0.4, 2, 4, and 7.7 m depth, respectively. The mean MAA concentration in the filamentous cyanobacteria decreased with depth from 1.32 at 0.4 m to 0.238 µg/µg Chl‐*a* at 7.7 m depth, whereas the underlying epilithic community decreased from 0.01 to 0.0005 µg/µg Chl‐*a* for the same depths.

### 3.4 Temporal dynamics of MAAs

**FIGURE 3:**
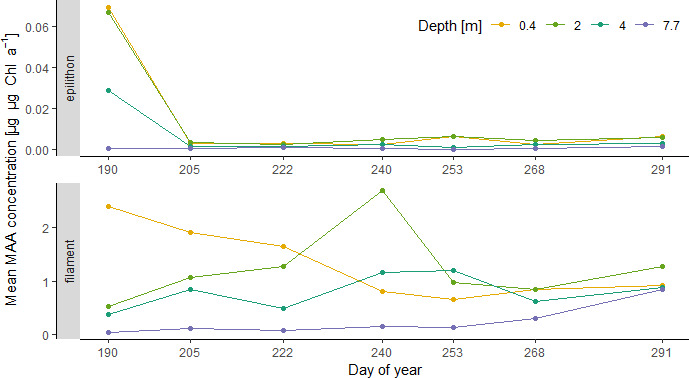
Temporal dynamics of mycosporine‐like amino acid (MAA) concentration in cyanobacterial filaments (filament, lower panel) and in the epilithic community (epilithon, upper panel) for the study period between 7 July and 18 October 2018 and the four depths [Colour figure can be viewed at wileyonlinelibrary.com]

Throughout the ice‐free season, average Chl‐*a*‐specific MAA concentrations for all depths in the underlying epilithon were very low (Figure 3) and ranged from 0.04 µg/µg Chl‐*a* on 9 July (doy 190) to 0.004 µg/µg Chl‐a on 18 October (doy 291). The same data, but for the filamentous cyanobacteria, however, showed higher concentrations and fluctuations without a clear temporal pattern (Figures 3 and S16). The concentrations ranged from 0.66 (doy 268) to 1.2 µg/µg Chl‐*a* (doy 240) and were negatively correlated with depth (*r* = −0.65, *p* < 0.01, *n* = 28). A Kruskal–Wallis test (χ^2^ = 38.166, *p* < 0.05, *df* = 1) corroborated the significantly lower concentrations in the underlying epilithic community than in the filamentous cyanobacteria. Overall, MAA concentrations of the underlying epilithic community showed only very low values with exception of the first sampling date, where they reached up to 0.070 µg/µg Chl‐*a* in 0.4 m depth (Figure 3). Mycosporine‐like amino acid concentration in the filamentous cyanobacteria fluctuated in all depths with the maximum found on different days depending on the depth considered (i.e. on doy 190 at 0.4 m, doy 240 at 2 m, on doy 253 at 4 m, and on doy 291 at 7.7 m depth, Figure 3). On the first three sampling dates, MAA concentrations clearly decreased with depth. This pattern, however, changed on doy 240, but was found again on the last two sampling dates. On doy 240, the MAA concentration in 0.4 m decreased to a value below the concentration in 2.0 and 4.0 m depth. Mean MAA concentrations in filamentous cyanobacteria were significantly correlated with water temperature (*r* = 0.58, *p* < 0.01, *n* = 28), but not with PAR levels (*r* = 0.28, *p* = 0.077).

## 4 DISCUSSION

Cyanobacterial assemblages are usually the dominant members of the benthic primary producer community in alpine lakes (Mez et al., 1998; Rott & Pernegger, 1994; Vinebrooke & Leavitt, 1996), as well as in other cold aquatic habitats such as those found in the Arctic and Antarctica (Quesada et al., 1999; Vincent et al., 1993). The taxonomic composition of the cyanobacterial community in our study site has been studied based on microscopic analyses (Rott & Pernegger, 1994). These authors described a clear depth zonation with a red‐brown *Gleocapsa* zone in the upper littoral (up to 0.3 m depth), where *Gleocapsa tornensis* is dominant and *Schizothrix* and *Calothrix* are found only in low abundance. This upper littoral area is subjected to the shear stress of the ice during winter and therefore cyanobacterial biomass is low and patchy. Then, a brown *Schizothrix* zone (0.5–2.5 m depth) with *Schizothrix simplicior* is found in exposed areas, but with *Tolipothrix* dominating in sheltered areas. The zone between 2.5 and 5.5 m depth is dominated by *Tolipothrix*, but with inclusions of *Nostoc*, *Calothrix*, *Dichothrixm, Phormidium, Plectonema*, and *Schizothrix*. In this depth zone and during the ice‐free season, a massive development of the filamentous green algae *Spirogyra* was described by the authors, but it was not observed during our study. Finally, the deepest zone (>5.5 m) is characterised by *Nostoc* (Rott & Pernegger, 1994). As discussed by the authors, the identification of many of these taxa is rather difficult because of their morphological variability. Further, since the publication of this study, cyanobacterial taxonomy underwent several revisions, thus raising concerns about the correct genus or species attribution. We attempted a comparison between microscopic analyses done by one of those authors (E. Rott) and molecular DNA analysis; however, this gave a poor comparability (data not shown). Therefore, we cannot link our results on the occurrence of the novel MAAs to a particular taxon as we also did not attempt to separate the entangled filament network they form.

The benthic community in general is shielded from exposure to solar UVR for many months by the presence of an ice/snow cover, but especially during early summer, radiation levels reaching even the bottom of these clear lakes can be high. Thus, the synthesis and accumulation of MAAs in cyanobacteria constitute one of the first lines of defence (Fuentes‐Tristan et al., 2019; Gao & Garcia‐Pichel, 2011; Hartmann et al., 2015; Rastogi et al., 2014; Whitelam & Codd, 1986) together with that of scytonemin (Garcia‐Pichel & Castenholz, 1991). Another adaptation known for benthic cyanobacteria is the migration into deeper levels in mat‐forming communities (Bebout & Garcia‐Pichel, 1995; Cockell & Knowland, 1999; Gao & Garcia‐Pichel, 2011); however, this is not feasible for epilithic communities growing on a thin biofilm.

Despite the intensive research on MAAs in cyanobacteria since the 1990s, we were able to identify a novel representative of this compound class. Due to the large biomass needed to isolate and structurally elucidate the MAAs and elucidate by NMR, we were able to determine the structure of the most dominant of the three unknown MAAs. Regardless of the large differences in taxonomic composition among the depth zones sampled, this MAA (λ_max_ = 334 nm, Figure 1) was dominant in almost all samples. This novel MAA had the highest similarity with AP‐C, which is known from the sea hare *Aplysia californica* (Kamio et al., 2011), but differed in the position of the methyl group which was attached to the nitrogen of C3 instead of C1. In this marine invertebrate, the three APs (AP‐A, AP‐B, and AP‐C) are apparently obtained through their algal diet and for AP‐A and AP‐B an alarm cue function has been proposed (Kamio et al., 2011; Kicklighter et al., 2011). Aplysiapalythine‐A and AP‐B have been also found in several seaweeds (Orfanoudaki et al., 2019) and AP‐C was tentatively identified in red seaweeds (Jofre et al., 2020).

The other two unknown and possibly new MAAs present in the samples were linked to a hexose, thus they are putatively mycosporine alanine hexose (retention time: 14.9 min) and mycosporine serine hexose (retention time 10.6 min). This agrees with the fact that cyanobacteria and fungi are the most common organisms reported to synthesise MAA glycosides (D’Agostino et al., 2016; Wada et al., 2015). Interestingly, this type of MAAs shows radical scavenging activity, for example in *Nostoc commune* (Matsui et al., 2011).

As expected, Chl‐*a*‐specific MAA concentrations in benthic cyanobacterial communities of Gossenköllesee significantly changed along the depth gradient. This is presumably due to changes in light (UV) conditions, although a significant correlation was only found with water temperature that co‐correlates with the light levels. This probably can be explained by the fact that temperature values change less than light levels during the day, and even if we used daily integrated values, it is unknown how the different time scales of light history affect the synthesis of MAAs. In any case, the negative correlation between depth and MAA concentration is consistent with previous research on planktonic communities (Tartarotti & Sommaruga, 2006) and benthic mats (Bonilla et al., 2005). These studies showed that communities exposed to higher UV levels synthesise higher concentrations of sunscreen compounds. Nevertheless, a strong seasonality of MAA concentrations in the epilithon or in the cyanobacterial filaments, as known for phytoplankton (Tartarotti & Sommaruga, 2006), was not found.

Very few studies have investigated the concentration of MAAs in benthic algal communities, but our measured mean Chl‐*a*‐specific MAA maximum (1.32 µg/µg Chl‐*a*) was comparable with that found for cyanobacterial mats in intertidal mangrove flats of Australia (Karsten et al., 1998). Perhaps more relevant is the fact that there were significant differences in the concentration found between the loosely attached filamentous cyanobacteria and the underlying epilithic ones. In the latter, concentrations were similar to that of microbial mats in the high Arctic (Mueller et al., 2005), and this indicated that the shading caused by the on‐top filamentous cyanobacteria is sufficient to dampen their synthesis of MAAs. For example, in microbial mats with a thickness of up to 8 mm in the high Arctic, the bottom layer of those mats is largely shielded from exposure to UVR (Quesada et al., 1999). One could also argue that the filamentous cyanobacteria actually fully protect the epilithic community against potential deleterious effects of UVR, however, this needs to be tested.

In conclusion, our study revealed that benthic filamentous cyanobacteria shield the underlying epilithic algal community against negative UVR effects by shading, but also by synthesising MAAs that reduce the transmission of wavelengths within their window of absorption. The low temporal dynamics in MAAs concentration during the ice‐free season suggests that benthic cyanobacteria have probably a very low turnover regarding the synthesis of these secondary metabolites. This agrees with the results at least for *Nostoc* in Gossenköllesee, which has high biomass, but a very low turnover (Rott & Pernegger, 1994). Finally, our study highlights the need to use analytical methods other than HPLC such as NMR or ultra‐HPLC coupled to high‐resolution mass spectrometry (Geraldes et al.,  2020) to explore the large diversity of MAAs found in aquatic organisms.

## Supporting information

Figure S1

Figure S2

Figure S3

Figure S4

Figure S5

Figure S6

Figure S7

Figure S8

Figure S9

Figure S10

Figure S11

Figure S12

Figure S13

Figure S14

Figure S15

Figure S16

Figure S1‐16

## Data Availability

The data of the present study are available from the authors upon reasonable request.
