# Supplementary figures and images for: Low temporal dynamics of mycosporine‐like amino acids in benthic cyanobacteria from an alpine lake
Source: Freshw Biol. 2020 Oct 15;66(1):169–76. doi: 10.1111/fwb.13627 (PMC7821102; doi:10.1111/fwb.13627)

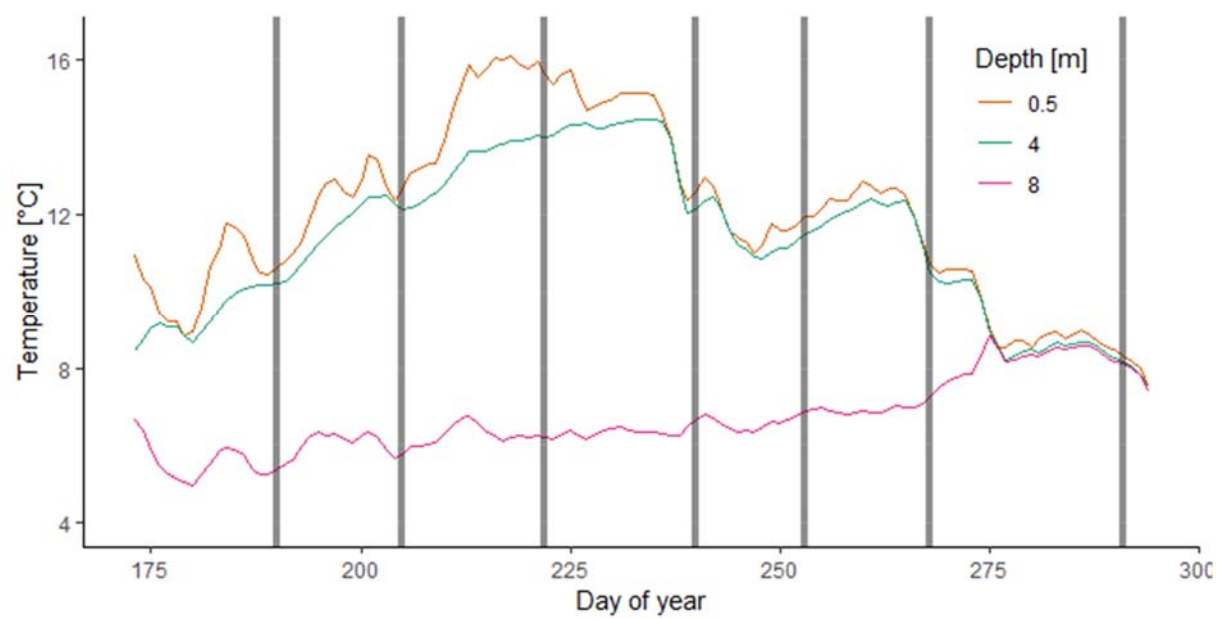

Supplement: Supplementary file 1 — Figure S1 [file FWB-66-169-s001.pdf]

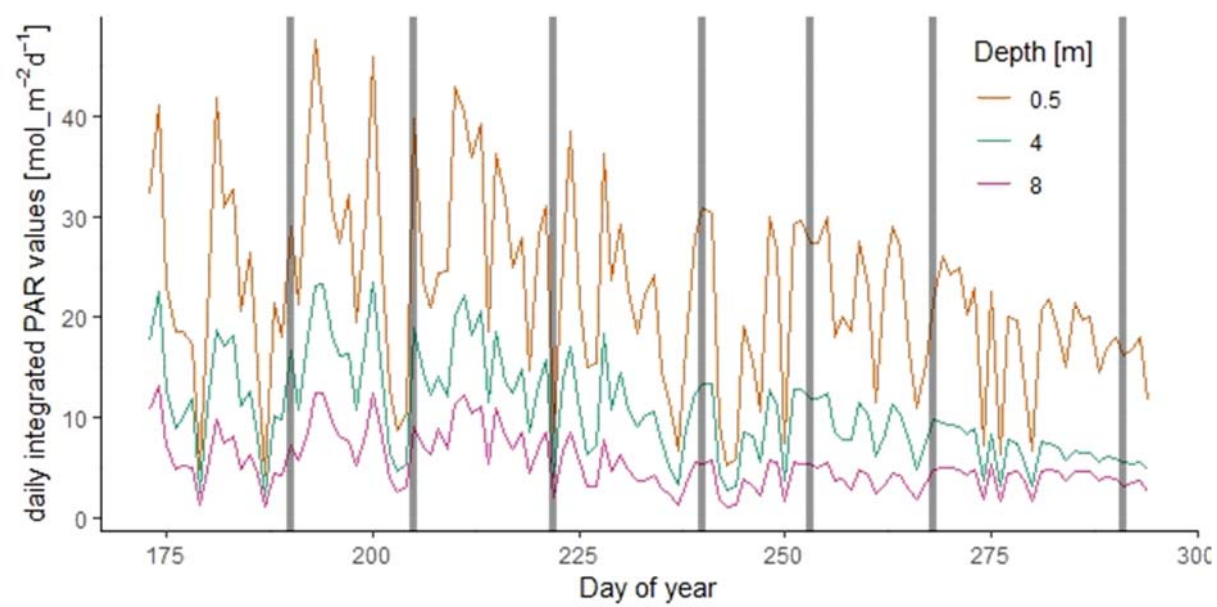

Supplement: Supplementary file 2 — Figure S2 [file FWB-66-169-s002.pdf]

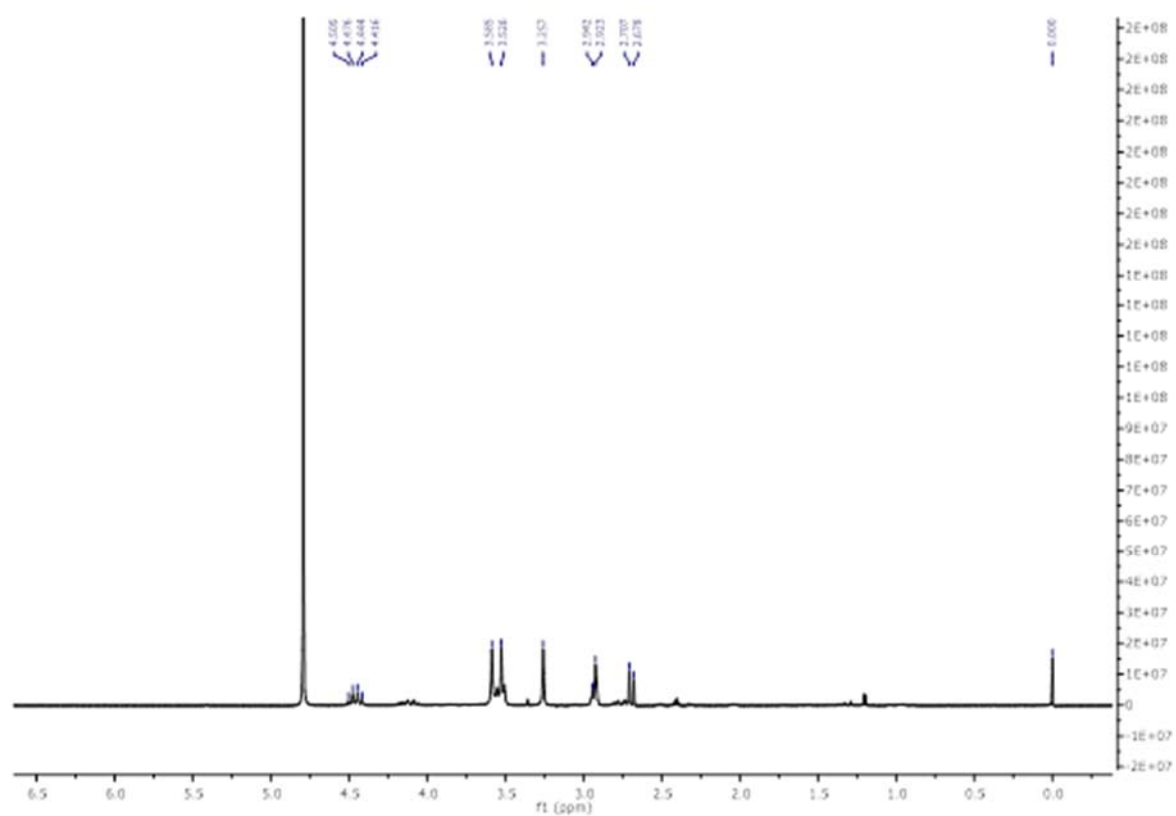

Supplement: Supplementary file 3 — Figure S3 [file FWB-66-169-s003.pdf]

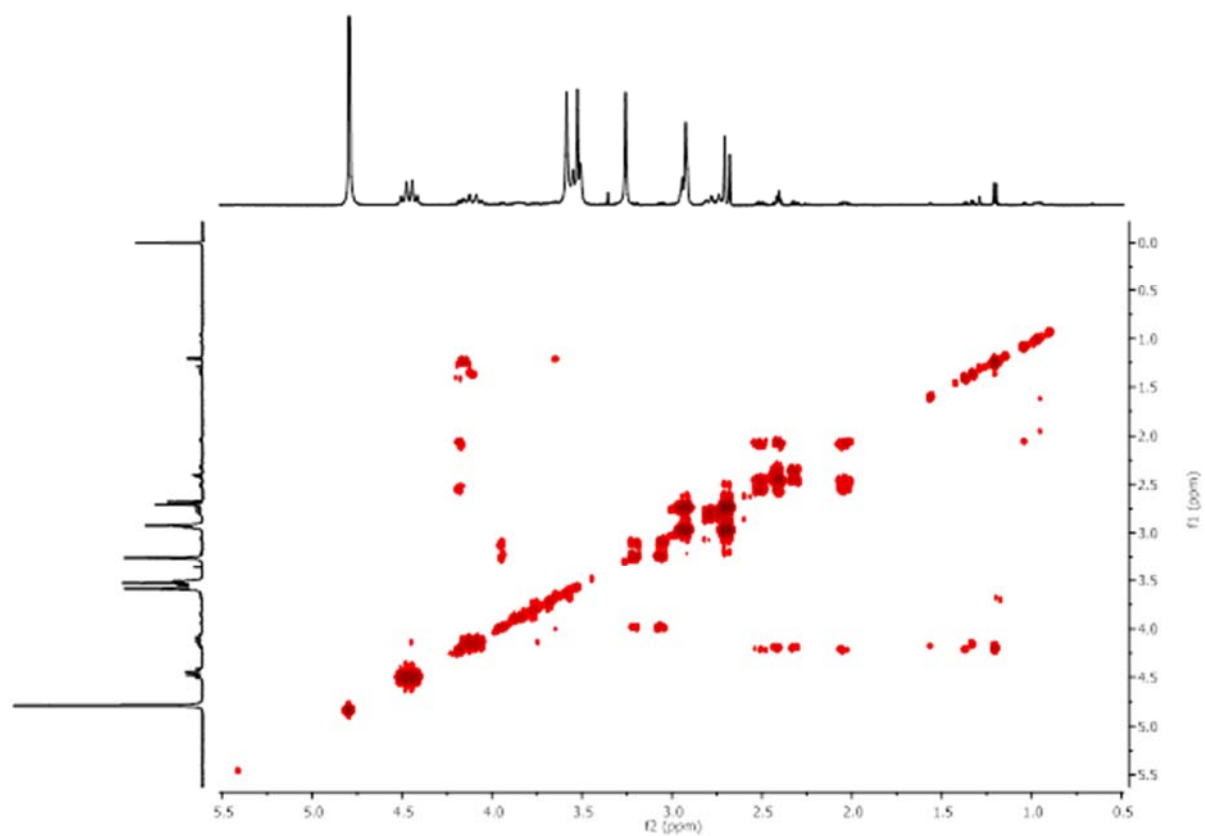

Supplement: Supplementary file 4 — Figure S4 [file FWB-66-169-s004.pdf]

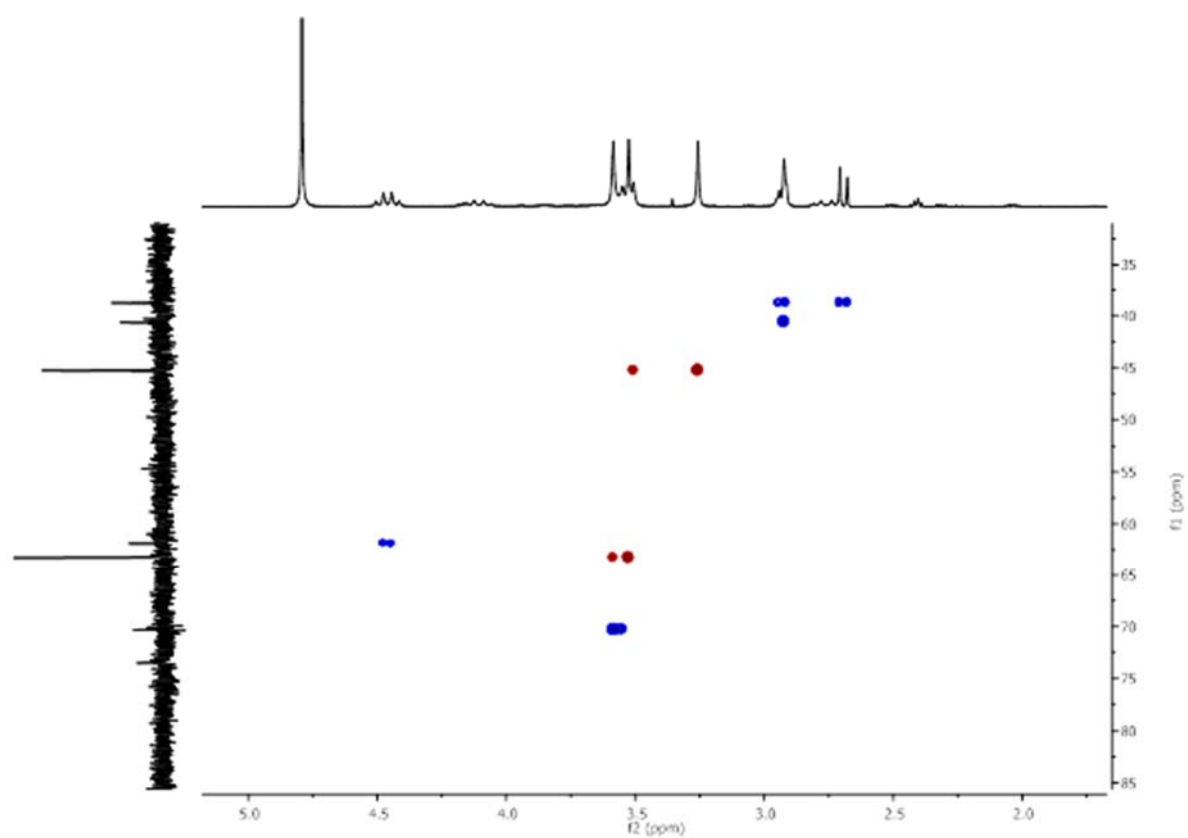

Supplement: Supplementary file 5 — Figure S5 [file FWB-66-169-s005.pdf]

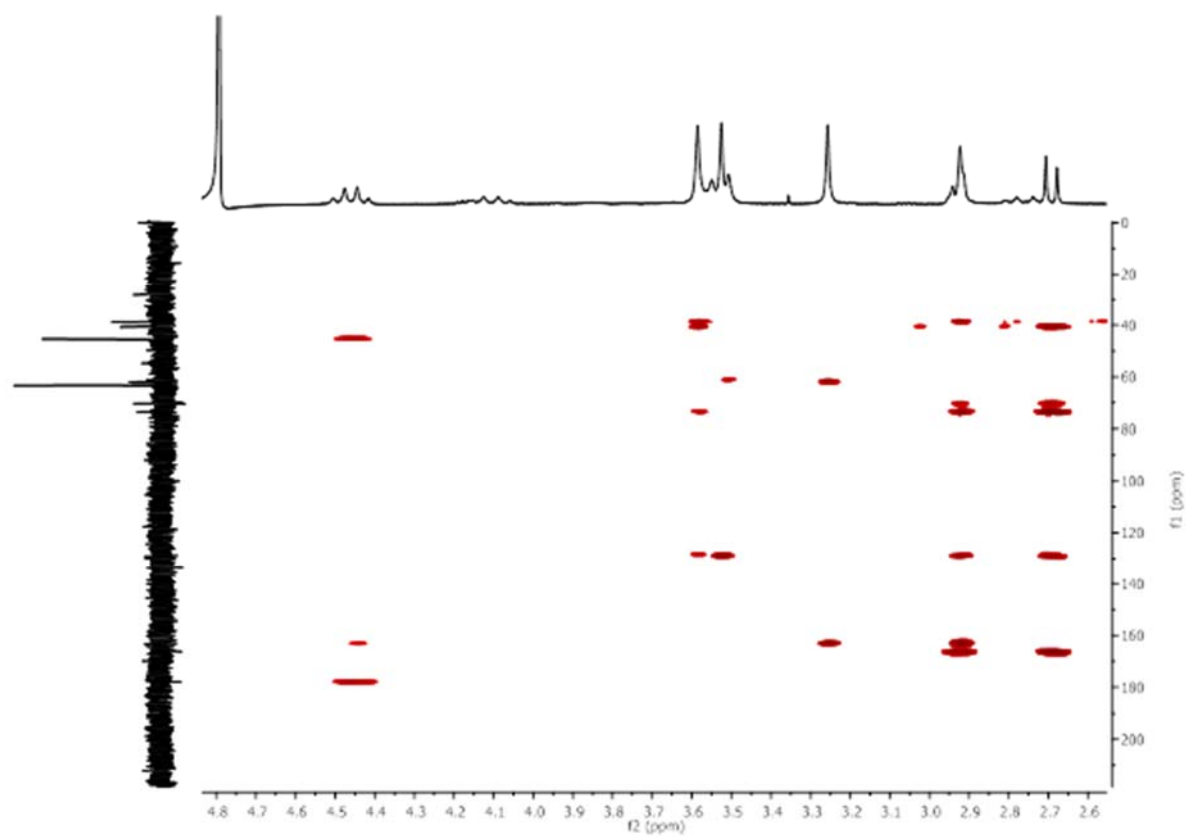

Supplement: Supplementary file 6 — Figure S6 [file FWB-66-169-s006.pdf]

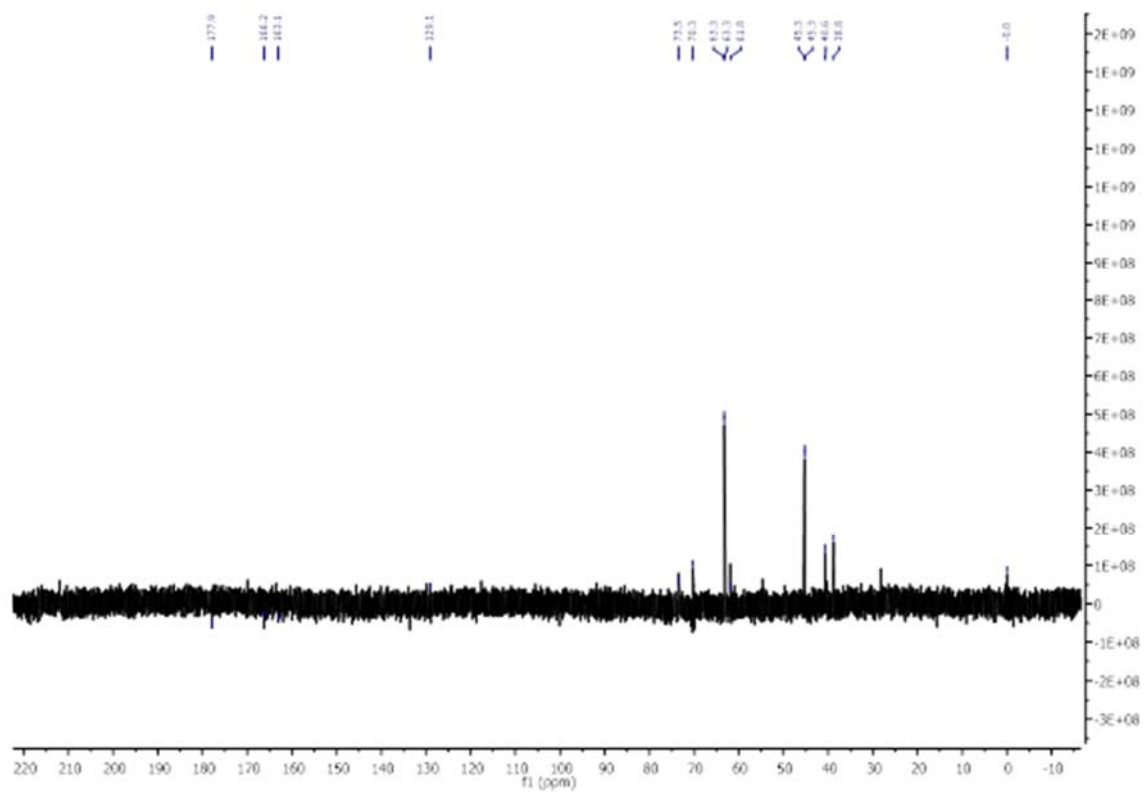

Supplement: Supplementary file 7 — Figure S7 [file FWB-66-169-s007.pdf]

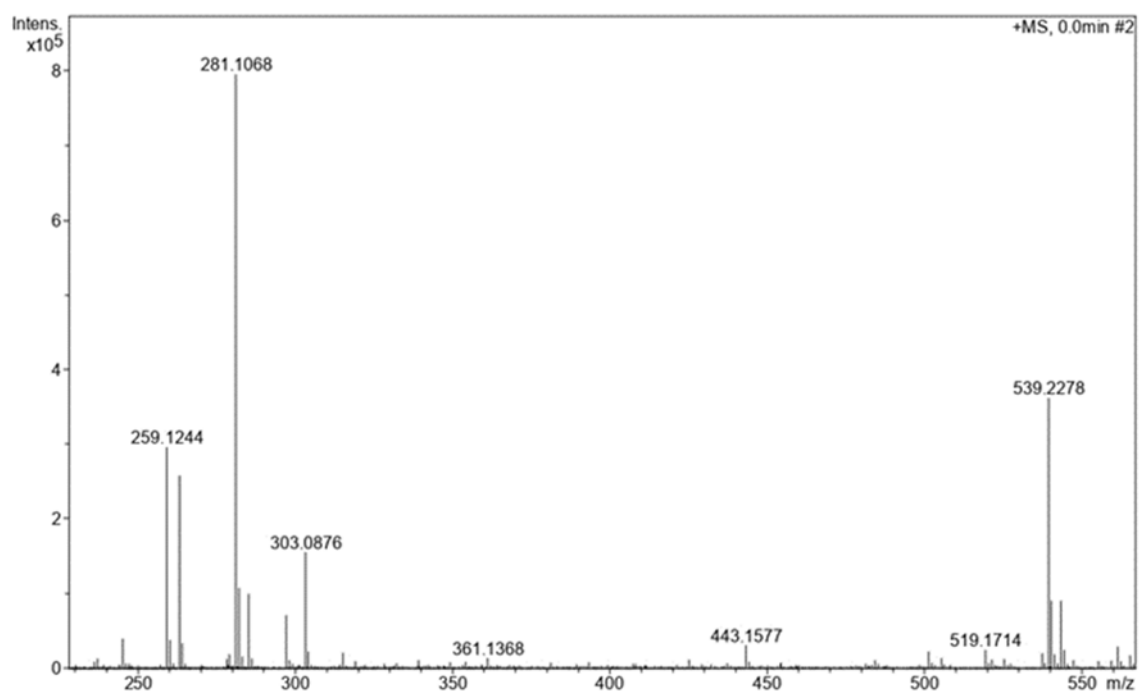

Supplement: Supplementary file 8 — Figure S8 [file FWB-66-169-s008.pdf]

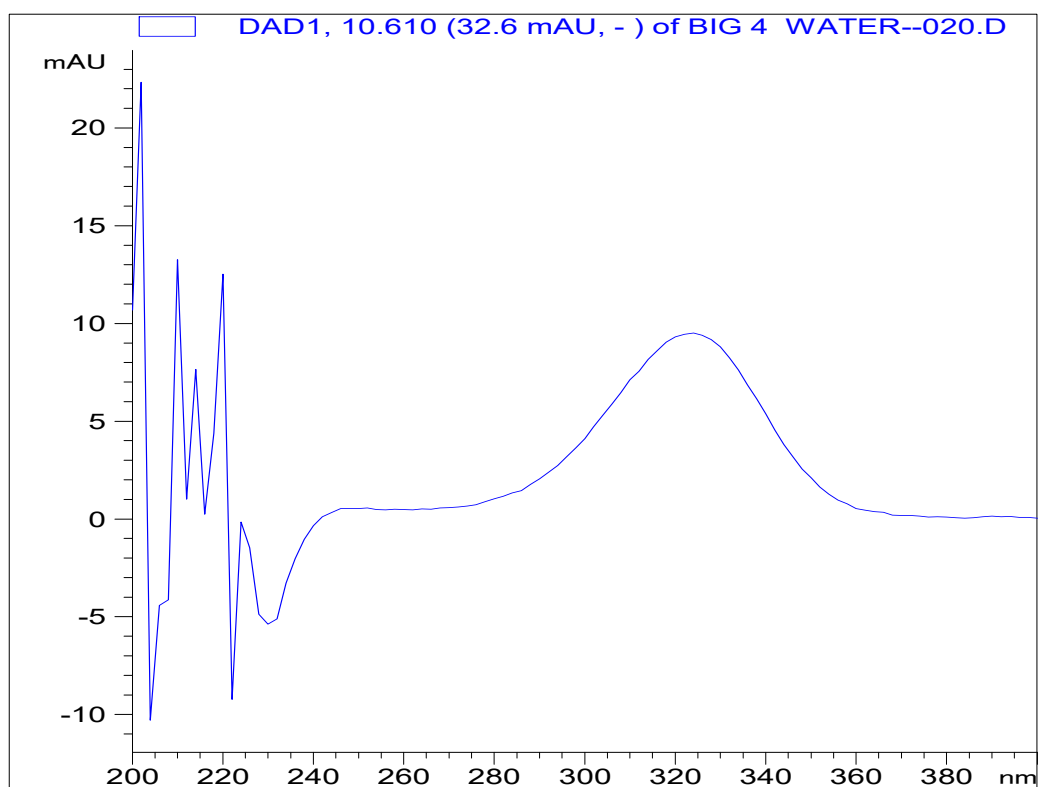

Supplement: Supplementary file 9 — Figure S9 [file FWB-66-169-s009.pdf]

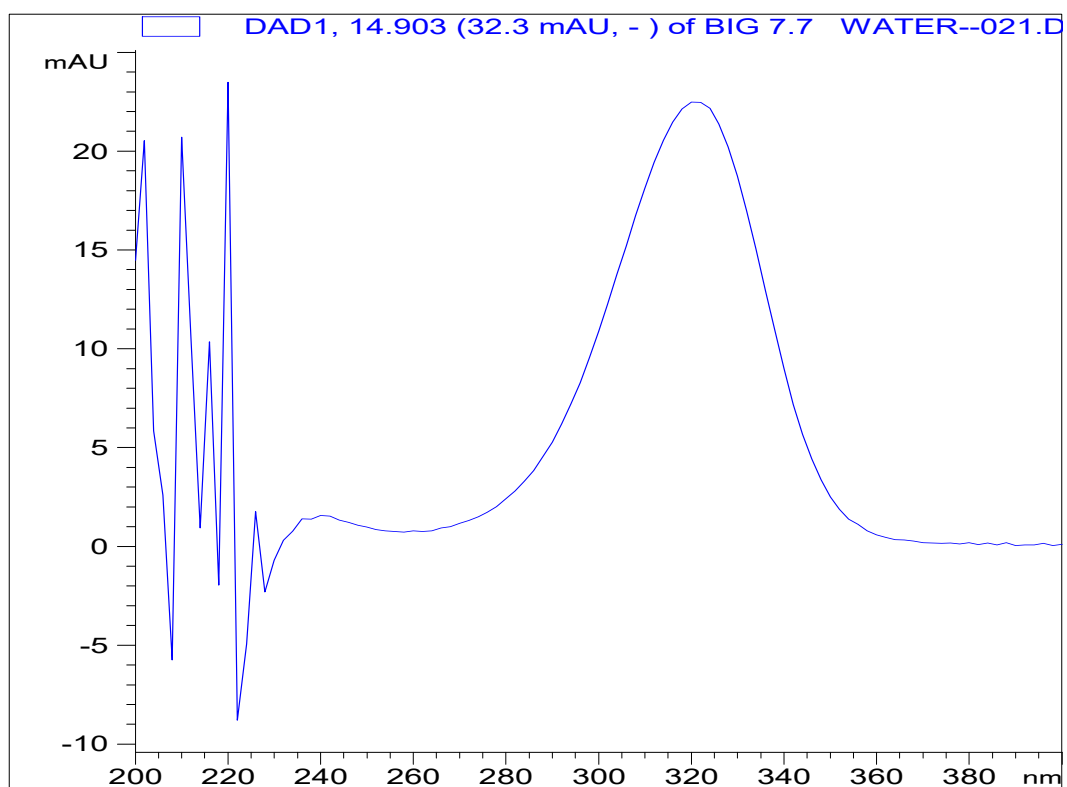

Supplement: Supplementary file 10 — Figure S10 [file FWB-66-169-s010.pdf]

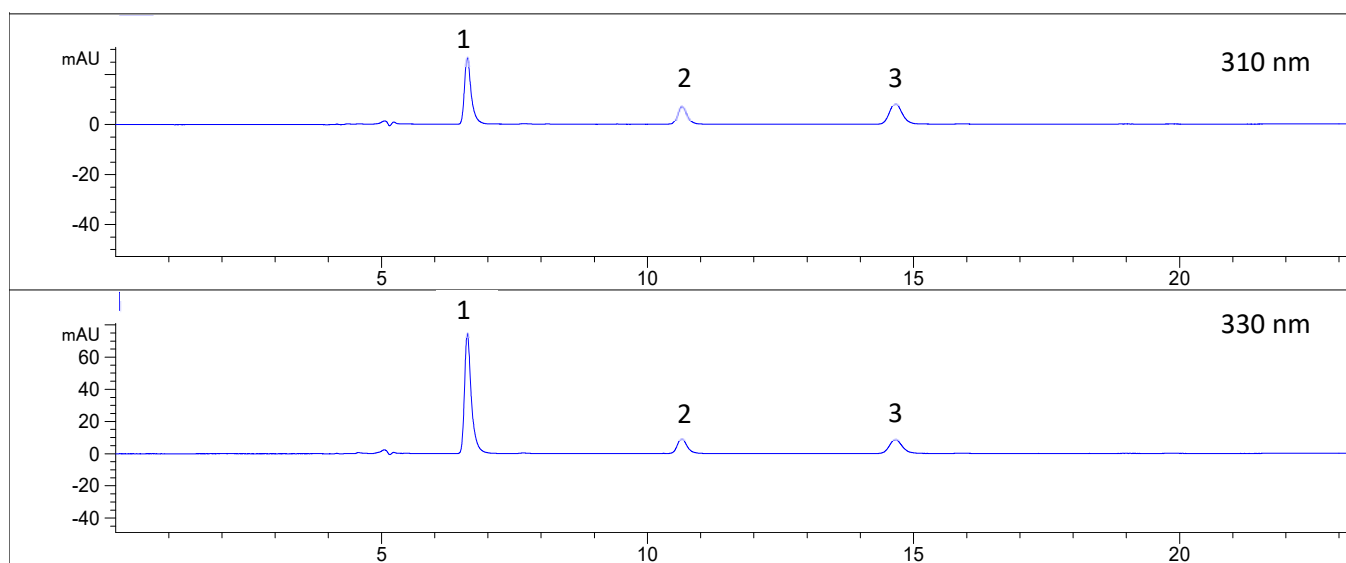

Supplement: Supplementary file 11 — Figure S11 [file FWB-66-169-s011.pdf]

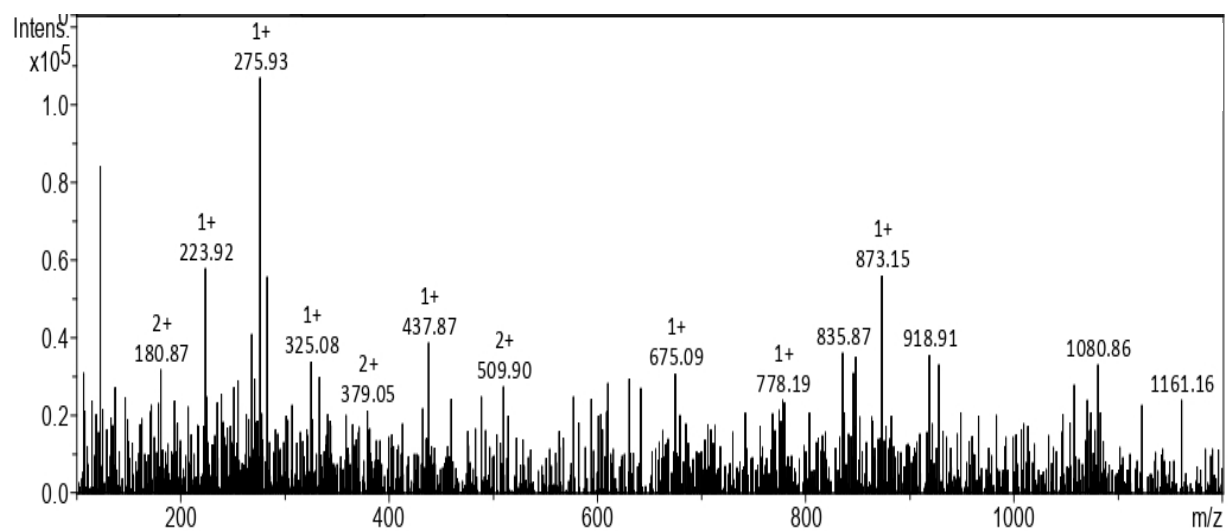

Supplement: Supplementary file 12 — Figure S12 [file FWB-66-169-s012.pdf]

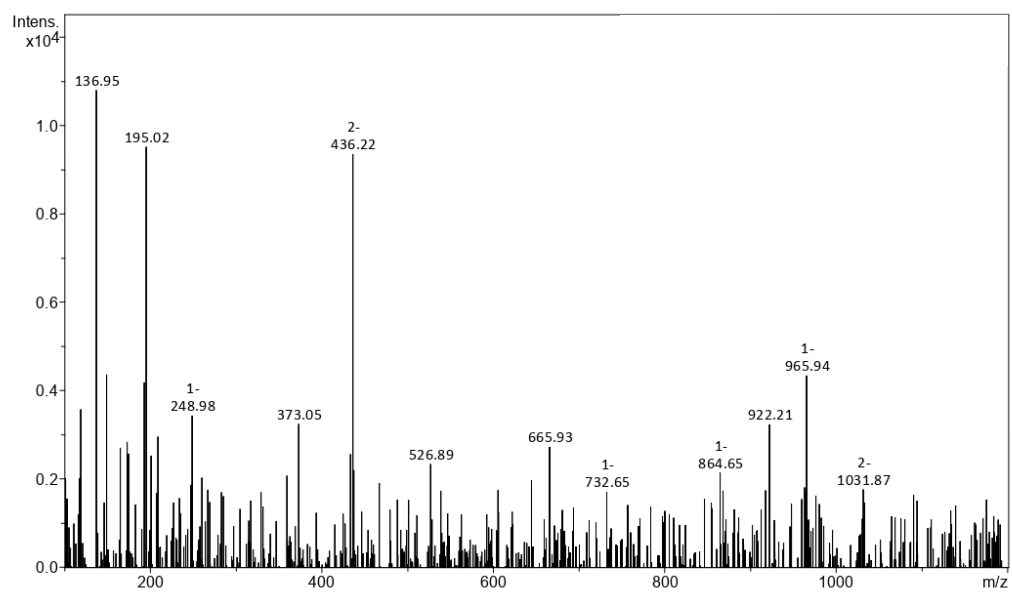

Supplement: Supplementary file 13 — Figure S13 [file FWB-66-169-s013.pdf]

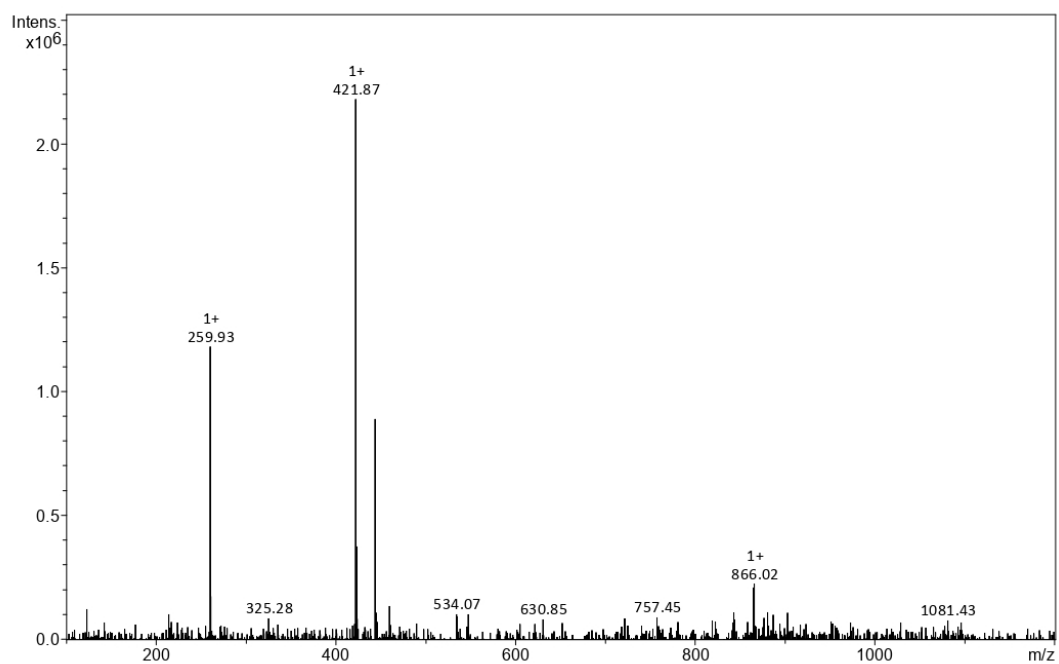

Supplement: Supplementary file 14 — Figure S14 [file FWB-66-169-s014.pdf]

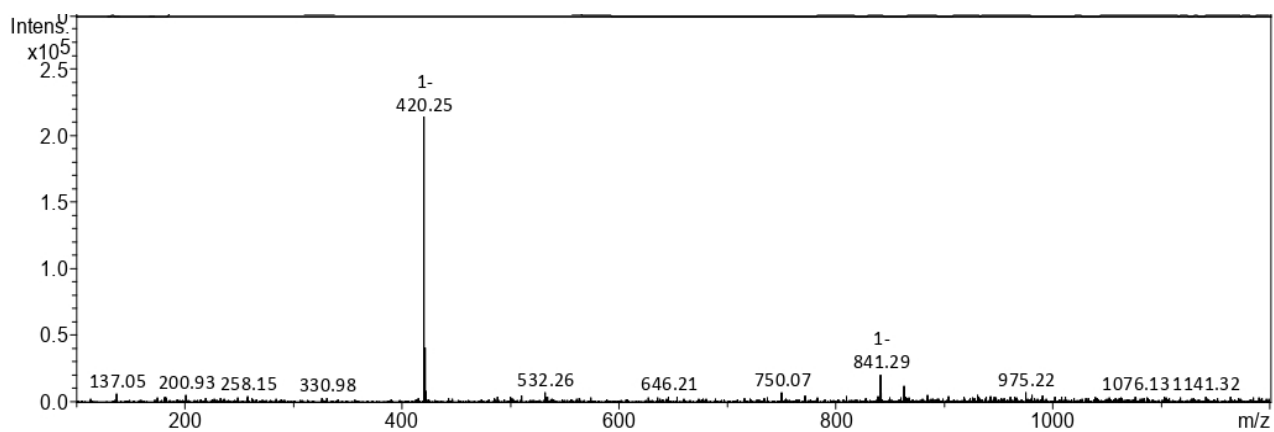

Supplement: Supplementary file 15 — Figure S15 [file FWB-66-169-s015.pdf]

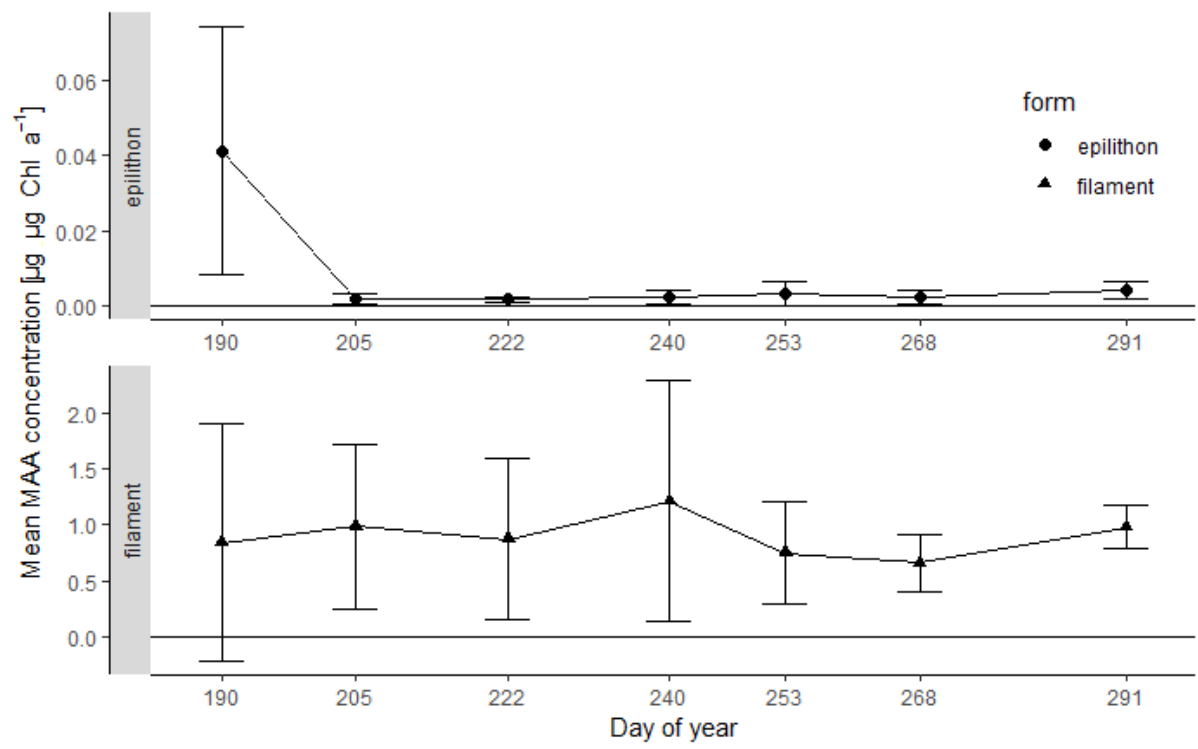

Supplement: Supplementary file 16 — Figure S16 [file FWB-66-169-s016.pdf]
