# Supplementary material for: Low temporal dynamics of mycosporine‐like amino acids in benthic cyanobacteria from an alpine lake
Source: Freshw Biol. 2020 Oct 15;66(1):169–76. doi: 10.1111/fwb.13627 (PMC7821102; doi:10.1111/fwb.13627)
Supplement: Supplementary file 17 — Figure S1‐16 [file FWB-66-169-s017.pdf]

Supporting Information for

Low temporal dynamics of mycosporine-like amino acids (MAAs) concentration in benthic cyanobacteria from an alpine lake

Nadine Werner[1] | Maria Orfanoudaki[2] | Anja Hartmann[2] | Markus Ganzera[2] | Ruben Sommaruga[1]

[1]Department of Ecology, University of Innsbruck, Technikerstr. 25, Innsbruck, Austria

[2]Department of Pharmacognosy, University of Innsbruck, University of Innsbruck, Innrain 80-82, Innsbruck, Austria

Correspondence: ruben.sommaruga@uibk.ac.at

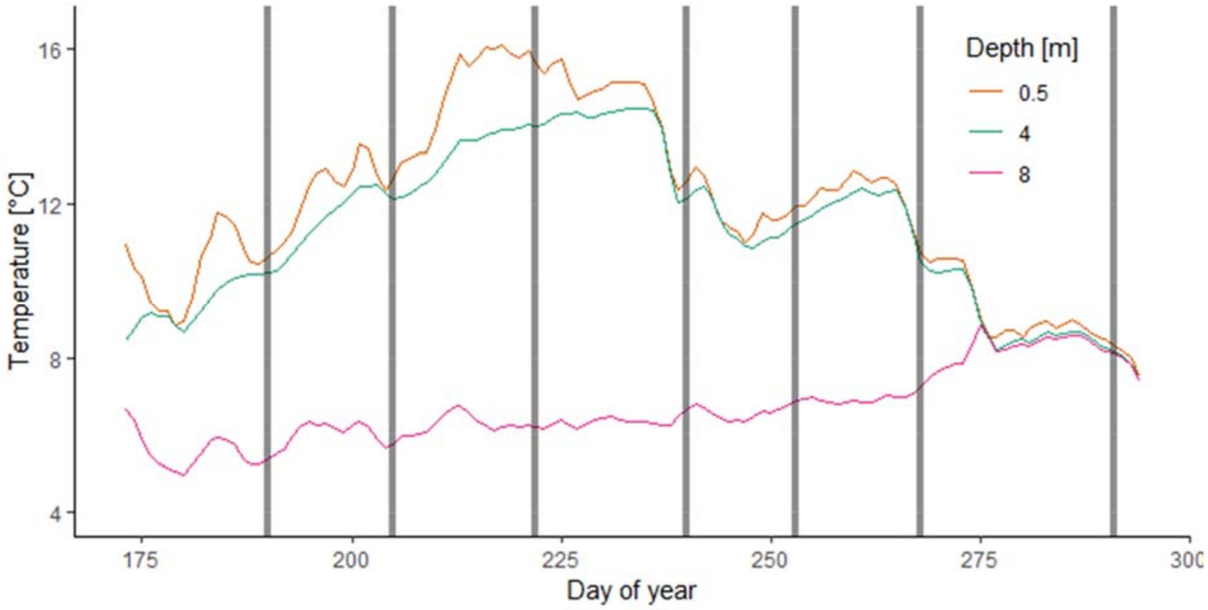

Figure S1. Daily mean temperature at 0.5, 4.0 and 8.0 m depth between June 22 and October 21, 2018. Grey vertical bars indicate sampling dates.

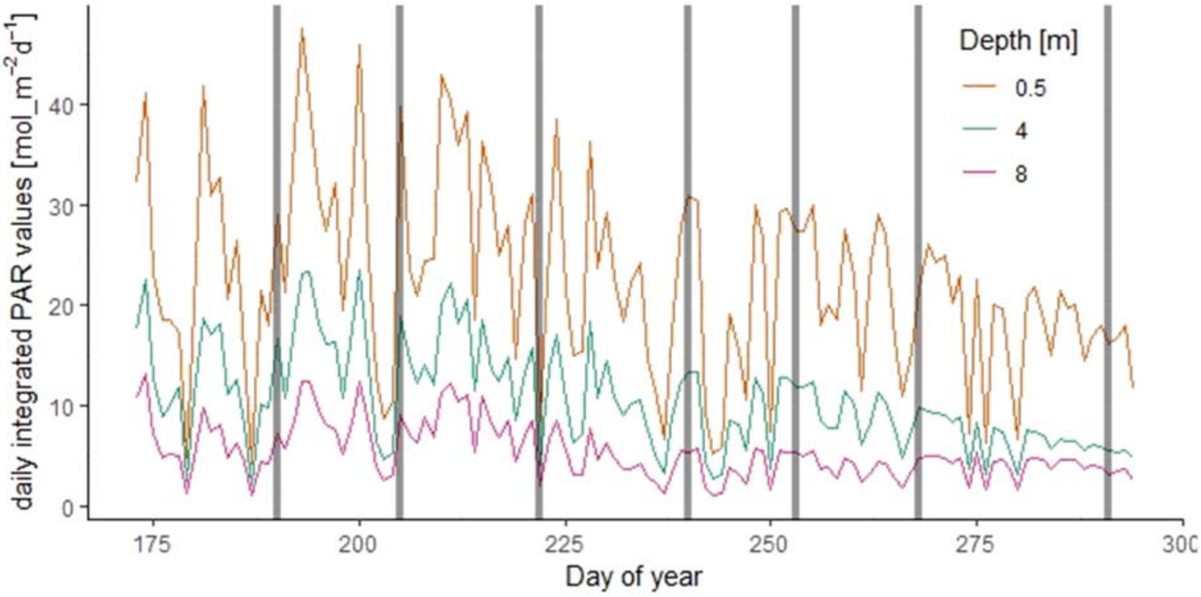

Figure S2. Daily integrated PAR values at 0.5, 4.0 and 8.0 m depth between June 22 and October 21, 2018. Grey vertical bars indicate sampling dates.

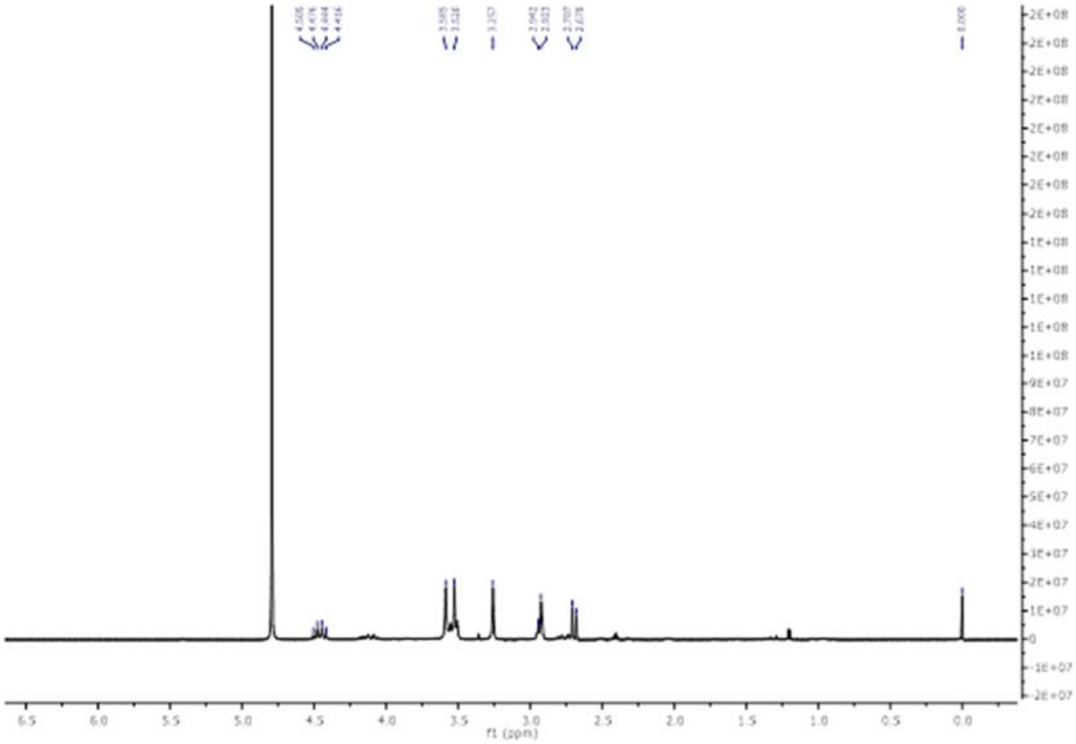

Figure S3. ^{1}H-NMR spectrum of the novel MAA in D_2O.

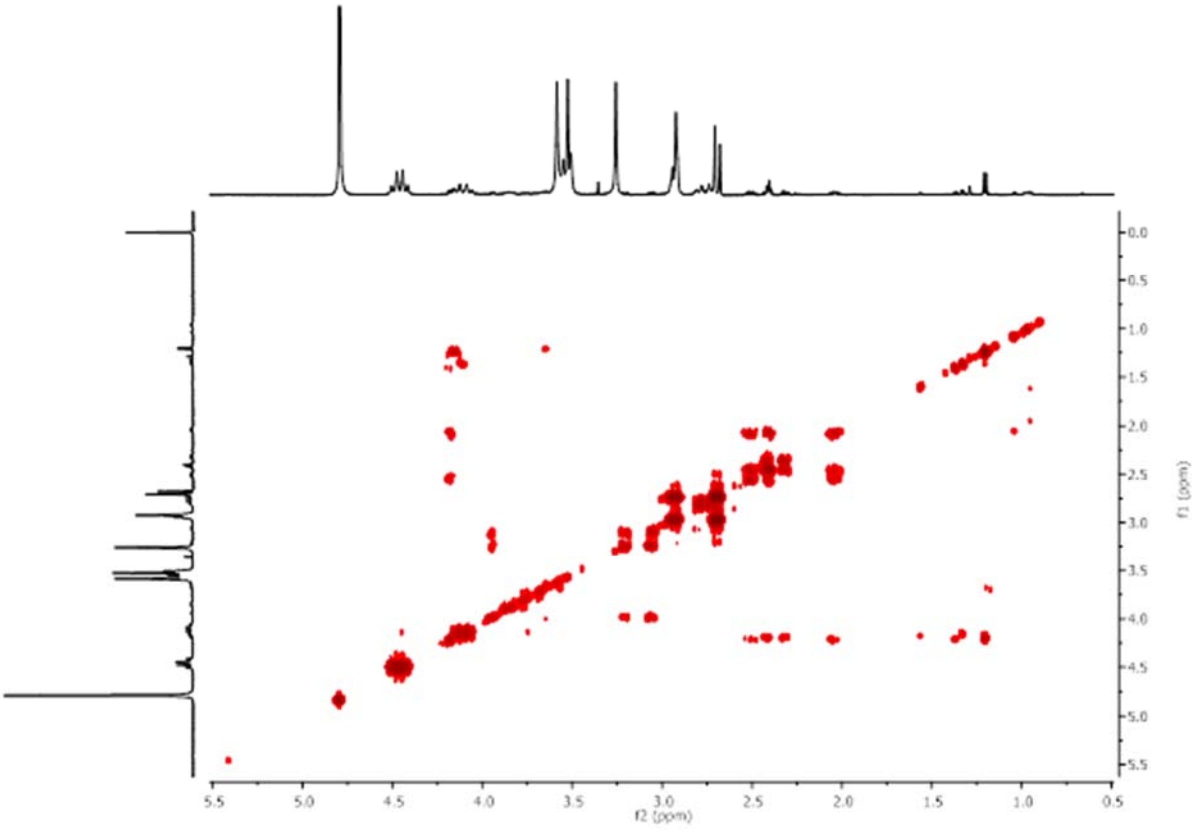

Figure S4. COSY spectrum of the novel MAA in D_2O.

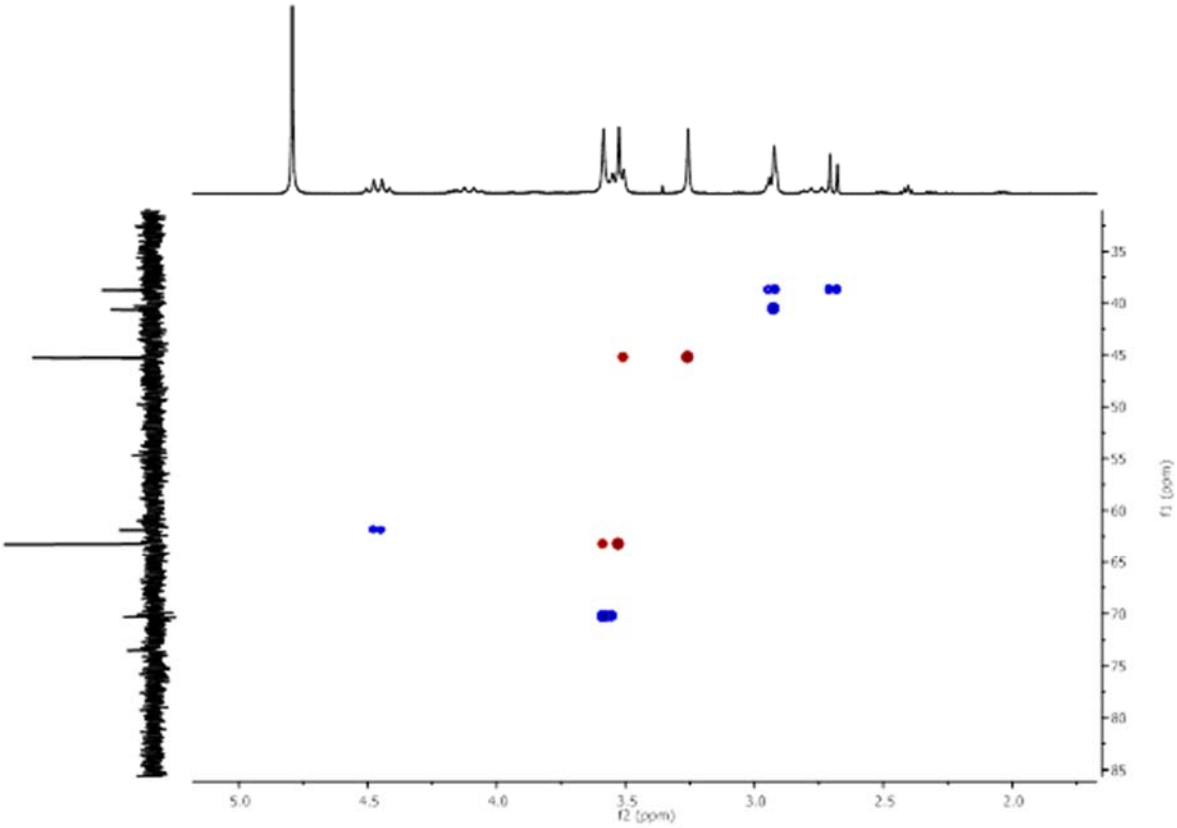

Figure S5. HSQC spectrum of the novel MAA in D_2O.

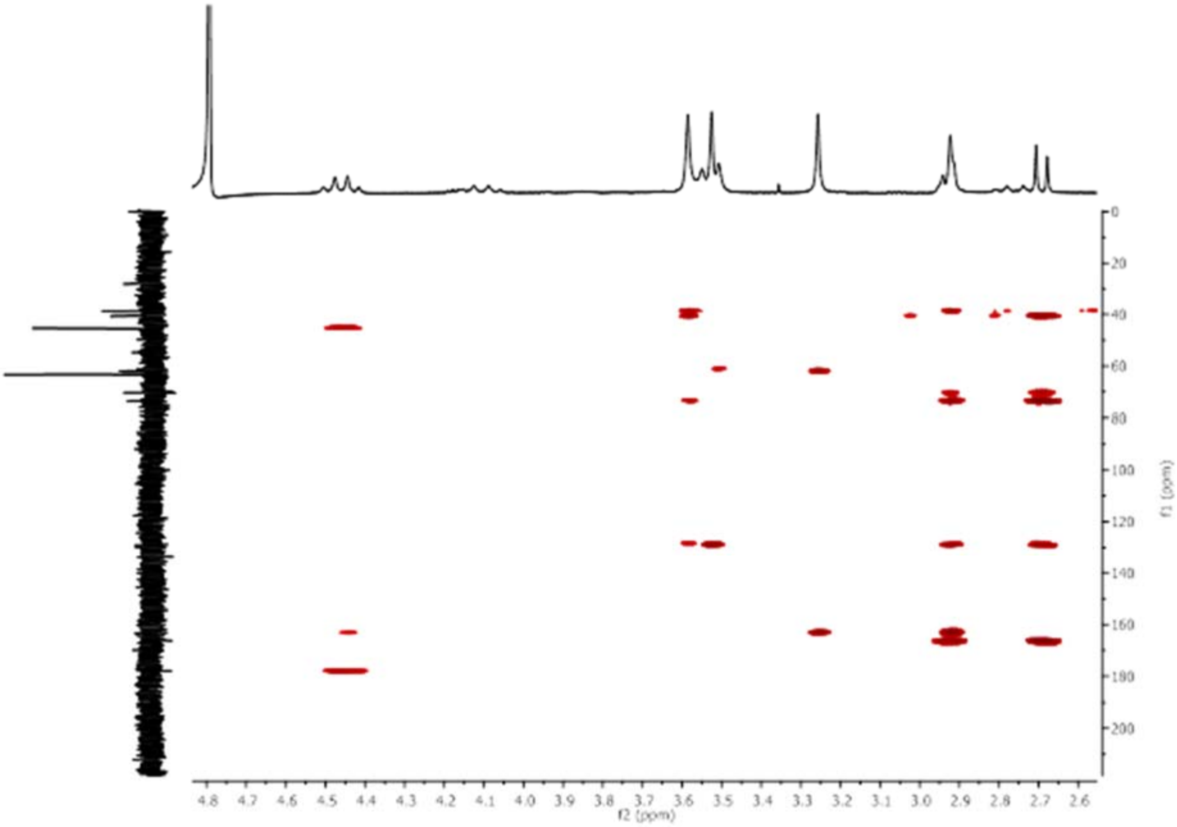

Figure S6. HMBC spectrum of the novel MAA in D_2O.

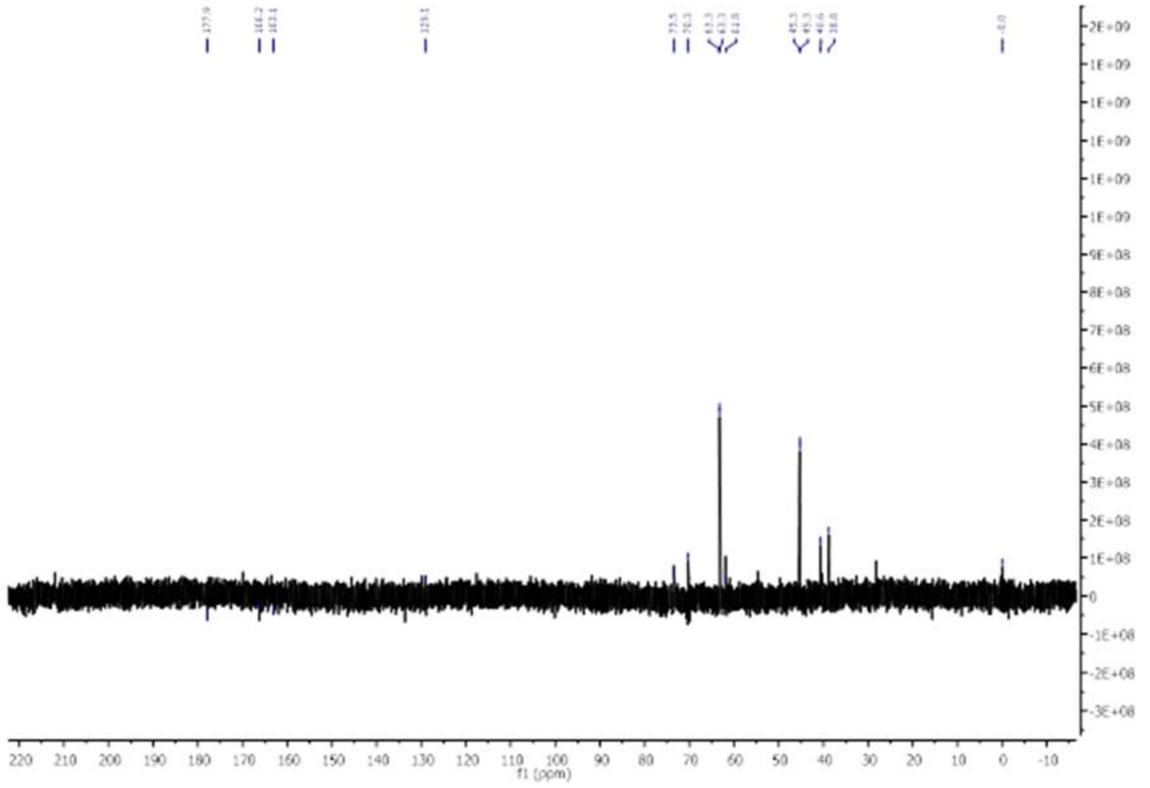

Figure S7. ^{13}C-NMR spectrum of the novel MAA in D_2O.

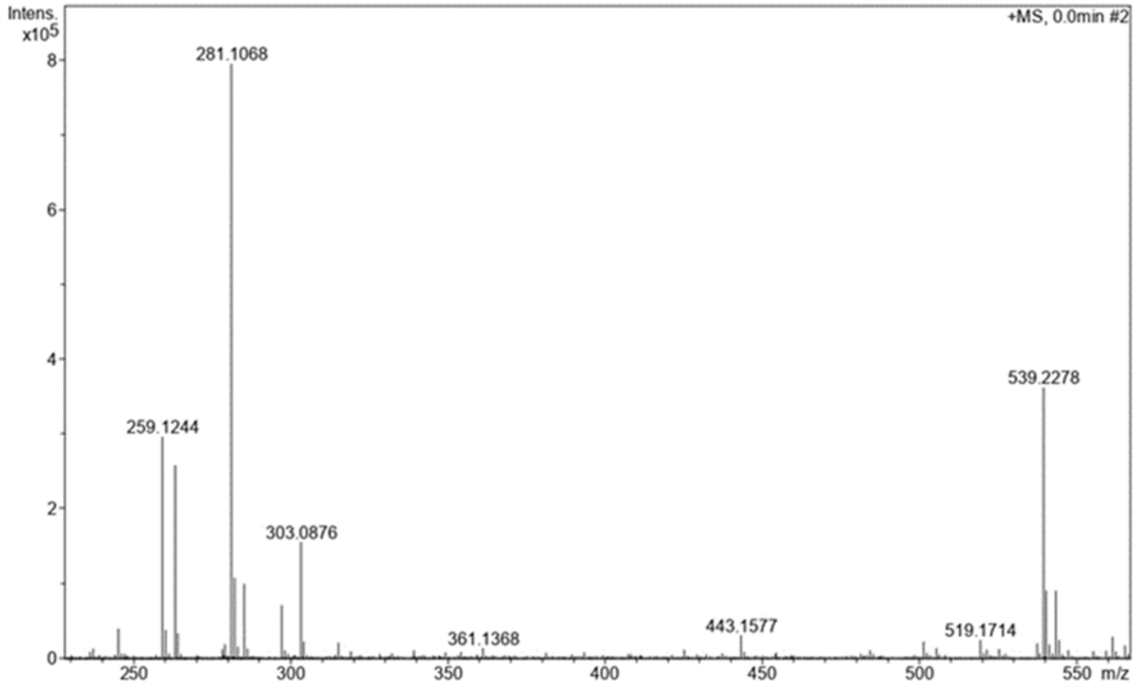

Figure S8. HRMS spectrum of the novel MAA (positive mode).

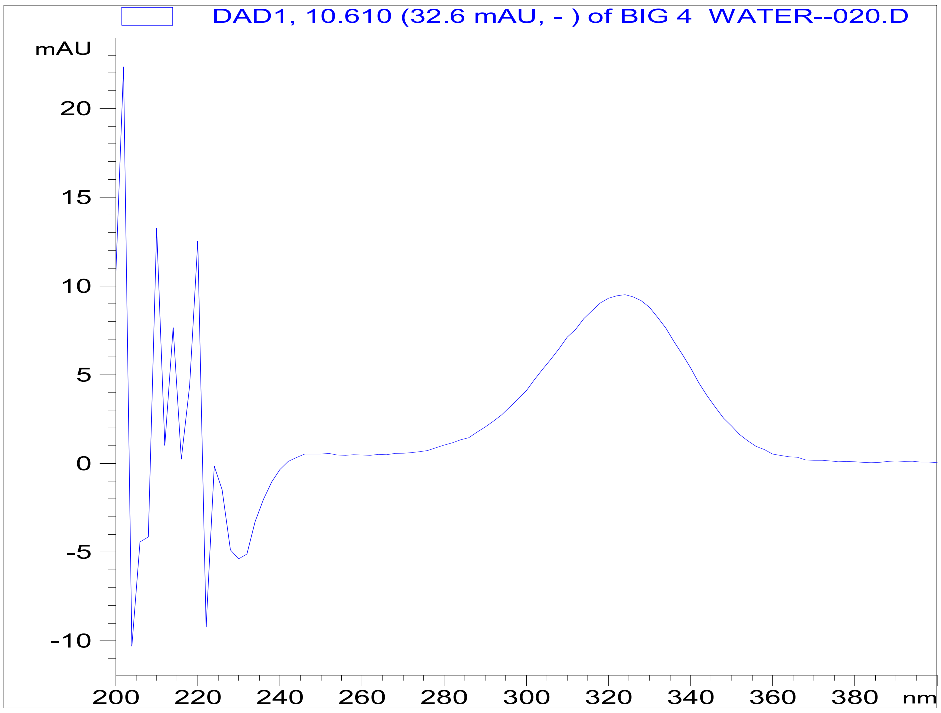

Figure S9. UV spectrum of the unidentified MAA at 10.6 min.

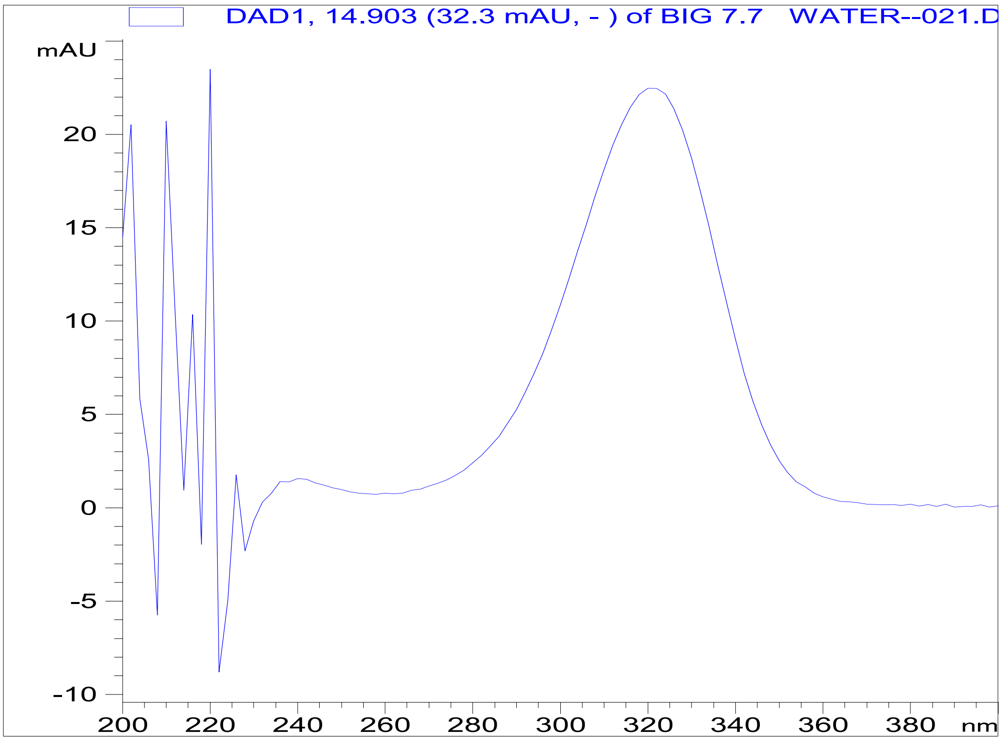

Figure S10. UV spectrum of the unidentified MAA at 14.9 min.

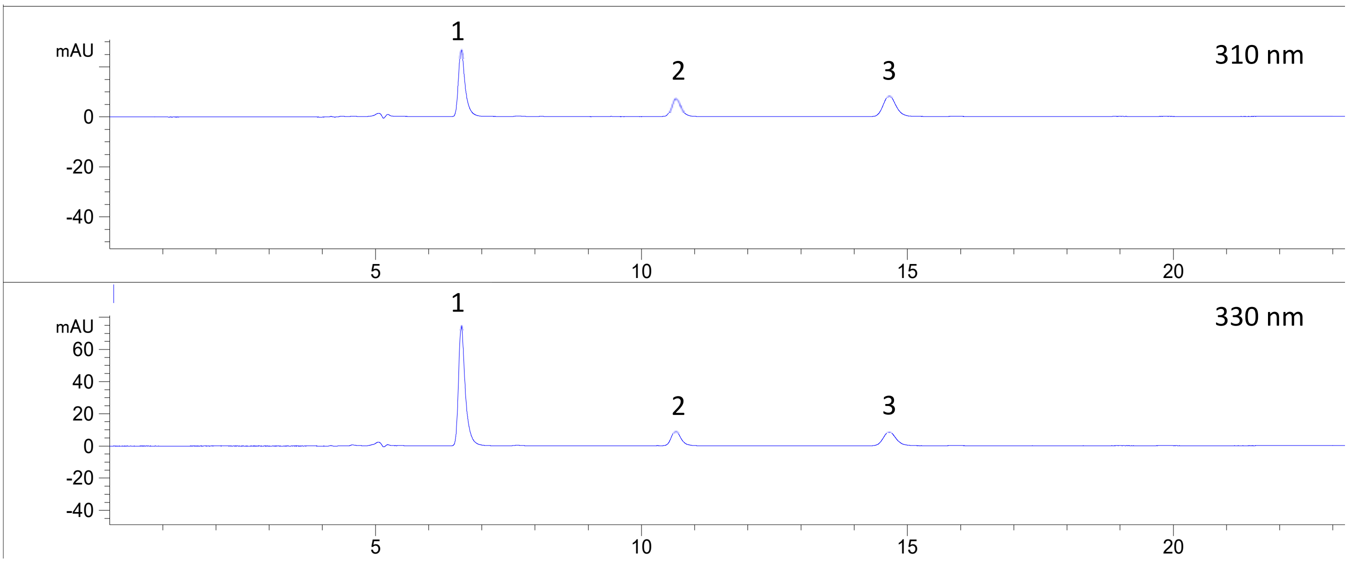

Figure S11. HPLC-UV separation of the extract. **1**: novel MAA, **2** and **3**: unidentified compounds; column: YMC-Pack ODS (250 x 4.60 mm, 5 µm), mobile phase: 20 mM ammonium formate and 0.25% (v/v) formic acid in water (A) and methanol (B); gradient: 0-15 min: 2% B, 23 min: 10% B, 30 min: 15% B, 35-40 min: 98% B, 40.1-50 min: 2% B; detection at 310 and 330 nm; flow rate=0.65 mL/min; T=20°C.

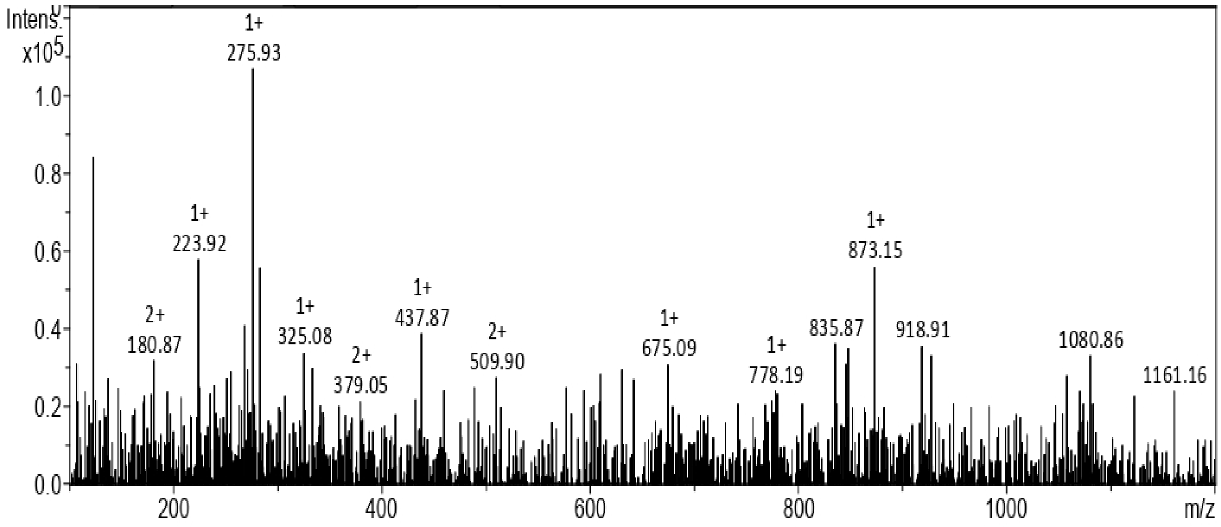

Figure S12. Mass spectrum of the unidentified MAA at 10.6 min (positive mode).

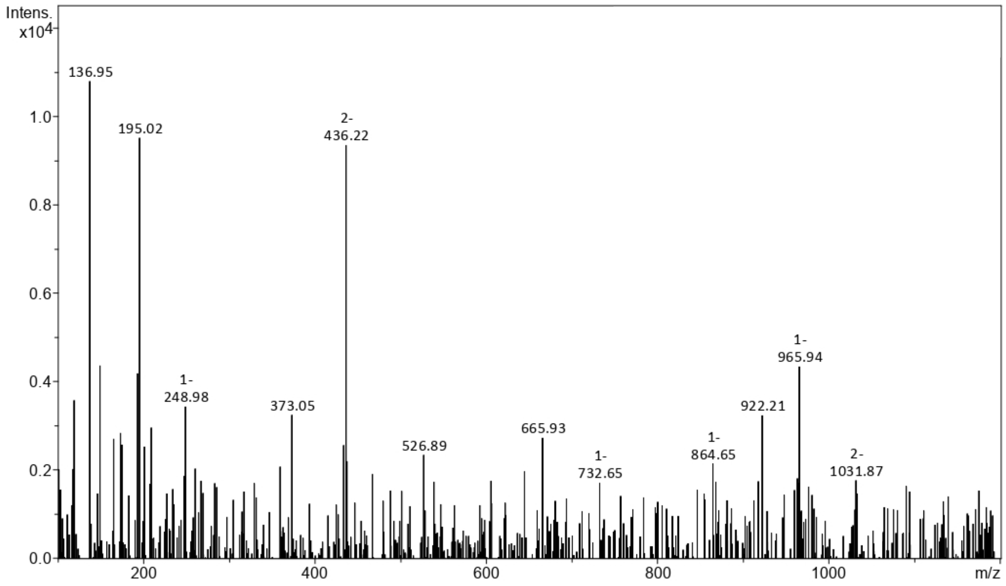

Figure S13. Mass spectrum of the unidentified MAA at 10.6 min (negative mode).

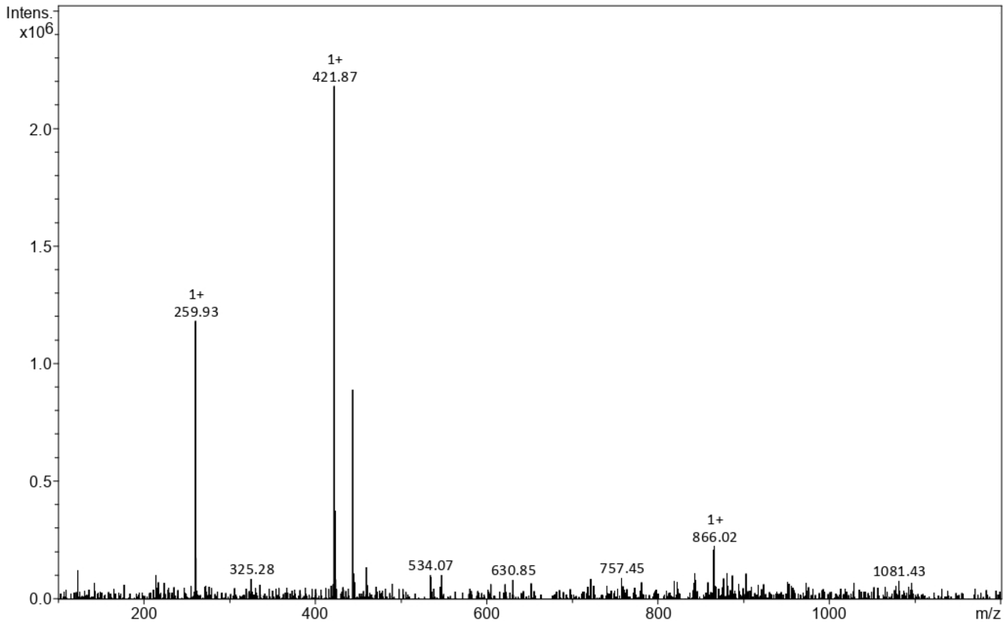

Figure S14. Mass spectrum of the unidentified MAA at 14.9 min (positive mode).

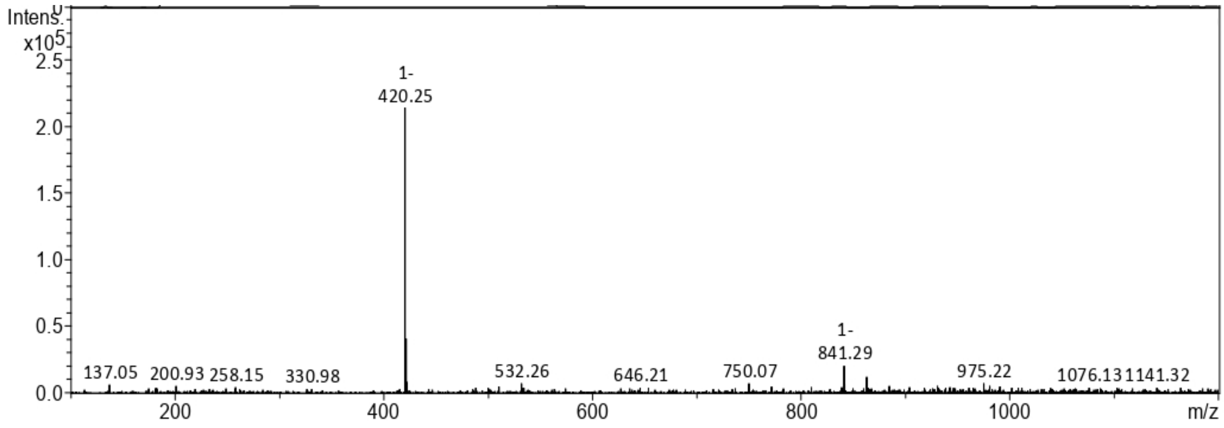

Figure S15. Mass spectrum of the unidentified MAA at 14.9 min (negative mode).

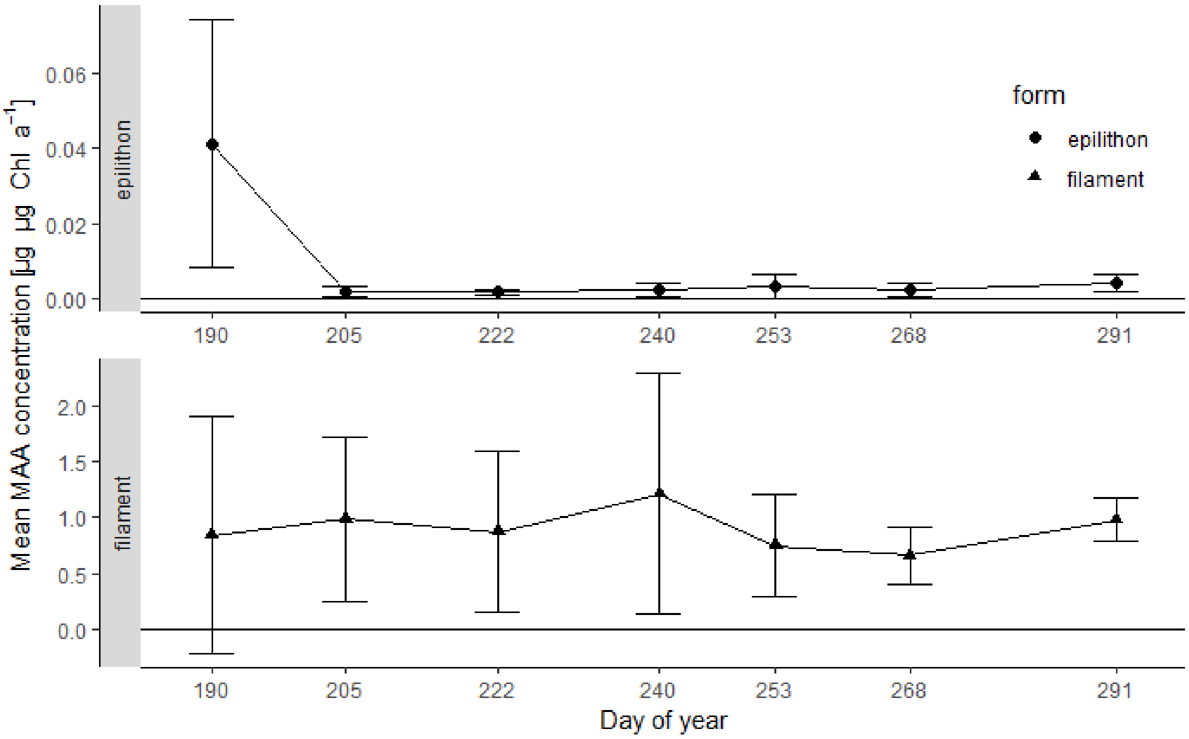

Figure S16. Mean Chl-*a*-specific MAA concentration for the study period between July 7 and October 18, 2018 in cyanobacterial filaments (filament) and in the epilithic community (epilithon). Values represent the mean ± 1 SD for all four depths.
